# Community support model on breastfeeding and complementary feeding practices in remote areas in Vietnam: implementation, cost, and effectiveness

**DOI:** 10.1186/s12939-021-01451-0

**Published:** 2021-05-17

**Authors:** Tuan T. Nguyen, Nemat Hajeebhoy, Jia Li, Chung T. Do, Roger Mathisen, Edward A. Frongillo

**Affiliations:** 1 Alive & Thrive, FHI 360, Hanoi, Vietnam; 2 Nutrition Section, UNICEF Nigeria, Abuja, Nigeria; 3 School of Business, Nanjing University of Information Science & Technology, Nanjing, China; 4 Arnold School of Public Health, University of South Carolina, Columbia, SC USA

**Keywords:** Community outreach, Community support groups, Infant and young child feeding, Nutrition training, Breastfeeding, Complementary feeding, Vietnam

## Abstract

**Background:**

Poor access to healthcare facilities and consequently nutrition counseling services hinders the uptake of recommended infant and young child feeding (IYCF) practices. To address these barriers and improve IYCF practices, Alive & Thrive (A&T) initiated community support groups in remote villages across nine provinces in Vietnam.

**Objective:**

This study examines the effectiveness of the support group model and related project costs for reaching underserved areas to improve IYCF practices.

**Methods:**

To evaluate the model’s implementation and project costs, we reviewed implementation guidelines, expenditure and coverage reports, monitoring data, and budgets for the nine provinces. To evaluate the model’s effectiveness, we used a 3-stage sampling method to conduct a cross-sectional survey from April to May 2014 in three provinces entailing interviewing mothers of children aged 0–23 months in communes with (intervention; *n* = 551) and without support groups (comparison; *n* = 559).

**Findings:**

*Coverage*: From November 2011 to November 2014, in partnership with the government, A&T supported training for 1513 facilitators and the establishing 801 IYCF support groups in 267 villages across nine provinces. During this period, facilitators provided ~ 166,000 meeting/support contacts with ~ 33,000 pregnant women and mothers with children aged 0–23 months in intervention villages. *Costs*: The average project costs for supporting the meetings, compensating village collaborators, and providing supportive supervision through staff in commune health stations were USD 5 per client and USD 1 per contact. After adding expenditures for training, supportive supervision, and additional administrative costs at central and provincial levels, the average project cost was USD 15 per client and USD 3 per contact. *Effectiveness:* Survey participants in intervention and comparison communes had similar maternal, child, and household characteristics. Multiple logistic regression models showed that living in intervention communes was associated with higher odds of early initiation of breastfeeding (OR: 1.7; 95% CI: 1.1, 2.7), exclusive breastfeeding from 0 to 5 months (OR: 12.5; 95% CI: 6.7, 23.4), no bottle feeding (OR: 2.69; 95% CI: 1.82, 3.99), and minimum acceptable diet (OR: 1.51; 95% CI: 0.98, 2.33) compared to those living in comparison communes.

**Conclusion:**

The IYCF support group model was effective in reaching populations residing in remote areas and likely contributed to improved IYCF practices. The study suggests that the model could be scaled up to promote equity in breastfeeding support.

**Supplementary Information:**

The online version contains supplementary material available at 10.1186/s12939-021-01451-0.

**Table:** 

This article is a part of the Interventions and policy approaches to promote equity in breastfeeding collection, guest-edited Pérez Escamilla, PhD and Mireya Vilar Compte, PhD	

## Introduction

Countries across the world are facing the triple burden of malnutrition, undernutrition (underweight, stunted or wasted), micronutrient deficiencies and overweight or obesity [1–3]. According to recent global nutrition reports, the world is off course to meet targets for childhood stunting, wasting, and overweight, yet have made some progress in exclusive breastfeeding and low birth weight [1, 3]. As countries develop economically, inequity in access to critical nutrition and health services is likely to increase [1].

Adequate nutrition is essential for the growth and development of infants and young children [4, 5]. Various interventions could be used to improve IYCF practices, including interpersonal communication and other support [4]. Facility-based interpersonal communication, in which clients visit health facilities to meet with counselors, is most useful in population-dense areas with more potential clients and convenient transportation [6]. Facility-based communication may be less practical in remote areas where populations are scattered and transportation to health centers is more challenging [6]. In these remote areas, reducing participation costs can improve accessibility, and using local languages as well as considering cultural norms can increase acceptability and better supply relevant information and support for recommended feeding practices to villages [6].

Data from a systematic review and meta-analysis show that the community-based support group model is effective in improving breastfeeding practices in low- and middle-income countries as well as high-income countries [7]. Namely, peer support helps to increase early initiation of breastfeeding, decrease prelacteal feeding, and increase exclusive breastfeeding for infants aged 3, 4 and 5 months in low- and middle-income countries [7]. However, the studies in this analysis primarily came from data obtained through one-on-one support via the phone and through home visits made in higher-income countries and urban areas. Remote and underserved communities have not been adequately covered in the literature, with the exception of a research report that evaluated the implementation, cost, and impact of the UNICEF’s Community Infant and Young Child Feeding Counseling Package from Nigeria [8].

In Vietnam, health indicators have improved in recent decades. For example, among children aged 0–4 years, the prevalence of underweight decreased from 36.7% in 1999 to 19.9% in 2008 to 12.8% in 2018; the prevalence of stunting decreased from 38.7% in 1999 to 32.6% in 2008 to 23.2% in 2018 [9]. Nutritional status and feeding practices vary substantially, however, by place of residence, socioeconomic status, and ethnicity [10, 11]. Compared to other areas, mountainous and remote areas in Vietnam have higher burdens of disease, less healthcare access with stronger social and structural barriers, and shortfalls in human, financial, and information resources [12, 13]. Alive & Thrive (A&T) tested the effectiveness of a community support model for providing IYCF support services in hard-to-reach areas [14].

Limited discussion exists on the cost and effectiveness of community-based interventions in the context of Vietnam. To address gaps in the literature, we conducted this analysis to examine the effectiveness of the support group model and financial resources used for this model to improve IYCF practices in Vietnam.

## Methods

### Setting and intervention

Vietnam is a lower-middle-income country in Southeast Asia with a population of more than 95 million; life expectancy at birth is 75 years and 36% of the country’s population lives in urban areas [15]. Each year, there are almost 1.6 million live births. The neonatal mortality rate is 11 per thousand live births, making up about 68% of the nation’s infant mortality [5]. The country has 63 provincial-level, 640 district-level, and 11,000 commune-level administrative areas [15]. Of the districts, 85 (13.3%) are classified as poor, mostly in remote areas [16]. Key challenges for recommended IYCF practices, child growth, and child development in remote areas are socio-cultural customs, poor economic conditions, and difficulties in accessing healthcare facilities [14].

**Fig. 1:**
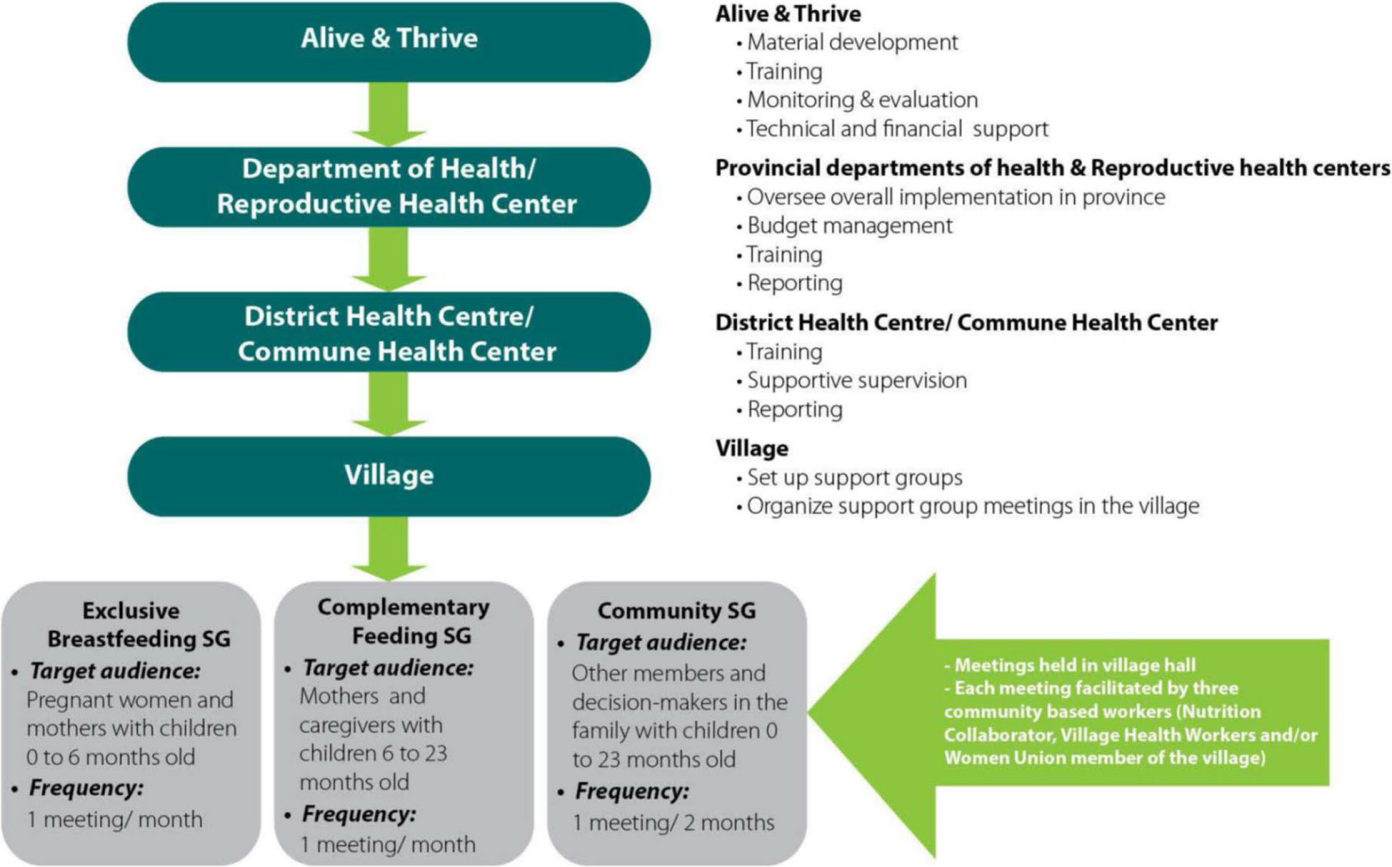
A&T support group model within the government health services

To address these challenges, A&T Vietnam collaborated with government health services starting in November 2011 to initiate 801 IYCF support groups in 267 villages within 78 remote communes across 9 provinces. Included were 12 communes in two poor districts, according to the active list of 61 poor districts from 2008 [17]. The IYCF support model was managed by commune health stations, supported by village heads, and implemented by facilitators **(**Fig. 1**)** [14]. The IYCF support group model became operational between November 2011 to September 2012, depending on provinces’ readiness; the model operated with A&T direct support until November 2014 until it became fully scaled-up and supported by local governments.

In each province, based on a list of criteria (Table 1), A&T staff worked with provincial, district, and commune health staff to select the villages to establish IYCF support group model and facilitators to lead the groups within the villages [14]. Facilitators, who were collaborators in health, population, nutrition, and within the Women Union, were trained on recommended IYCF practices, the use of job-aids and other materials, and skills to facilitate group discussions [14]. Provincial and district health trainers trained facilitators, while district and commune health staff provided supportive supervision.

**Table 1:** Support group and facilitator selection criteria [14]

Support group selection criteria	Support group facilitator criteria
• Local authorities are enthusiastic and committed to supporting IYCF practices. • Commune health staff, community-based workers, and women are active and enthusiastic about being involved in the project. • Health facilities are more than 5 km away from the village. • Local households live within a 2-km radius of the village’s central meeting area. • At least 10 pregnant women and mothers with children under two live in the village. • The stunting rate in the village is equal to or higher than national average (27%). • Poor IYCF practices are prevalent.	• Local person living in the village, familiar with the culture and participating mothers. • Speaks local dialect. • Lives in the village and is accepted by her/his community and health personnel. • Desires to learn and share experiences and knowledge with other members of the community. • Caring, considerate, and respectful. • Has time, energy, and support from family. • Open to learn good listening and communication skills, and effective question-posing skills (successfully demonstrated during training).

The support groups were informal and peer-led; facilitators lived in the villages and communicated in local dialects. Three groups were established in each village: Group 1 – Exclusive Breastfeeding Group, for pregnant women in the last trimester and lactating mothers with children aged 0–5 months; Group 2 – Complementary Feeding Group, for mothers and caregivers with children aged 6–23 months; and Group 3 – Community Support Group, for family members who influence feeding decisions in households with children aged 0–23 months (Fig. 1). Groups 1 and 2 met once a month and group 3 met once every 2 months. The hour-long support group meetings typically included 10–15 participants and occurred in a village hall. During the facilitated meetings, participants shared experiences, exchanged information, and supported each other. Fifteen topics were used in IYCF support groups: five for Group 1, seven for Group 2, and three for Group 3 (Table 2) [14].

**Table 2:** Topics of IYCF in support groups [14]

Groups	Topics
**Group 1.** Exclusive Breastfeeding Support Group	1) Nutrition during pregnancy and breastfeeding 2) Initiation of breastfeeding within one hour of birth 3) Exclusive breastfeeding in the first 6 months 4) Positioning and attachment of baby at breast 5) Preparation for complementary feeding
**Group 2.** Complementary Feeding Support Group	6) Feeding children, according to age, with a sufficient frequency of meals and sufficient amount and thickness of food each day 7) Feeding children diverse foods at each meal (4 food groups) 8) Preparation of a hygienic meal 9) Nutrition care for child during sickness (illness) and recovery 10) Nutrition care for child with diarrhea and acute respiratory infections 11) Responsive feeding – helping a child to eat well 12) Cooking demonstration
**Group 3.** Community Support Group	13) Causes and consequences of child malnutrition 14) Support from family and the community in breastfeeding 15) Support from family and the community in complementary feeding

In this study, we used desk reviews to estimate the effectiveness of the implementation, coverage, and cost, and a quasi-experimental cross-sectional design to evaluate the effectiveness of this intervention model.

### Desk reviews of the implementation, coverage, and cost

To evaluate the model’s implementation coverage and related project costs, we reviewed implementation guidelines and reports, monitoring data, and budget for the nine provinces.

The number of total meeting contacts and the unique number of pregnant women and mothers with children aged 0–23 months in intervention villages were obtained from the routine monitoring system [14] in each province. A&T introduced three forms for use by support group facilitators, including 1) the list of pregnant women and mothers with children aged 0–23 months (facilitator could use an alternative form from the Ministry of Health), 2) IYCF support group report to capture information on service delivery in the village for each of the three target groups, and 3) household visit record to keep track of home visits by each village collaborator **(**Additional file 1**)** [14]. Every month, the facilitators reported data on the meeting frequency, number of clients who participated in support groups by group type, and the number of pregnant women and children aged 0–5 and 6–23 months in each village to the manager of the commune health station. The manager and shared the report completed in Excel form (Additional file 1) with higher-level staff via email. A&T staff used the Pivot table function in Excel to manage, analyze, and present the data. Monitoring data demonstrated 1) the number of new clients (to estimate the unique number of clients), 2) total number of contacts by client and group type, and 3) coverage (total number of women who were pregnant or with a child aged 0–23 months and participated in group meeting divided by the number of women who were pregnant or with a child aged 0–23 months living in the villages) [14].

The cost of the IYCF support group model was estimated in the nine provinces from the perspective of a project sponsor, using mixed methods [18]. First, a bottom-up method was applied to estimate the cost of the following two categories: 1) delivery costs, including for meeting logistics, compensation of village collaborators, and supportive supervision by staff from commune health stations, and 2) training costs, including per diem for attending trainings, transportation, lodging, and compensation for lecturers. The costs of these two categories were estimated using actual time spent and cost per working hour of staff at the nine Provincial Departments of Health. The top-down method was applied to obtain the cost of the third category: other costs at the central and provincial levels, which included managing the project, meetings, communication, and reporting. The cost for the third category was estimated by subtracting the total cost (provided by the Finance and Contract Manager at A&T, covering the above items) from the sum of costs of the first two categories. From the perspective of the project sponsor, the three categories belonged to a one-time cost based on the amount of money paid by A&T.

For the unit cost estimation, the average cost per contact and per client was estimated by dividing the cost by the number of contacts and number of women obtained from the monitoring data. The key assumption for this estimation was that the IYCF support group functioned with the same level of efficiency and effectiveness across provinces, districts, and communes. We used tables in Excel to gather, manage, and analyze the costing estimation.

### Evaluation of model effectiveness of the IYCF support group model

To evaluate the model’s effectiveness, we used a quasi-experimental cross-sectional design with intervention and comparison communes at end-line only (i.e., with no baseline data and a non-random allocation to the intervention) [19].

#### Sampling

This cross-sectional survey was conducted in three provinces belonging to three Vietnam regions: Central (Quang Binh), Central Highland (Dak Nong), and Mekong Delta (Ca Mau). In each selected province, three-stage cluster sampling was used to select survey participants. Stage 1 – Select districts. We selected six out of seven districts with communes implementing IYCF support group models, the intervention, in the provinces. One district was excluded due to an insufficient number of pregnant women and children aged 0–23 months. This district’s four villages with IYCF support groups had a total of 9 pregnant women and 59 children aged 0–23 months, according to monitoring data from February 2014. In each province, two districts with similar characteristics were selected as the comparison districts. Stage 2 – Select communes. In each selected intervention district, two communes with IYCF support groups were randomly selected from the communes that had implemented the model. In each comparison district, two comparison communes without an IYCF support group were selected. The selected comparison communes were comparable with the intervention communes in terms of socio-economic status, malnutrition rates, and lack of nutrition interventions beyond the IYCF support groups implemented for this study. The comparison communes typically met criteria to participate in the IYCF support group model but were constrained due to A&T funding limitations. A total of 24 communes including 12 intervention communes and 12 comparison communes were selected from 3 provinces. Stage 3 – Selection of study participants. In each of the selected intervention and comparison communes, we used systematic random sampling to select 19 mothers with children aged 0–5 months and 27 mothers with children aged 6–23 months [20]. For sampling, we first used the list of all children aged 0–23 months and sorted it by date of birth (descending). Second, we identified the sampling integer (k) by dividing the number of children in the surveyed areas (N) by the sample required (n) for each of the two age groups (k = N / n). Lastly, we selected the random starting (r) from 1 to k, and then identified subsequent children by adding the first child code and the interval number (i.e., r, r + k, r + 2 k, etc.). In total, we interviewed 551 mothers of children aged 0–23 months in intervention and 559 in comparison communes.

#### Data collection

From April to May 2014, the survey was conducted by an independent research firm, the Institute of Social and Medical Studies (ISMS; Hanoi, Vietnam). Data were collected via face-to-face interviews with the use of a paper-based structured questionnaire that was developed by the A&T team and pretested several times in the field **(**Additional file 2**)**.

The team consisted of 18 data collectors (typically with a bachelor’s degree in public health, medicine, or sociology and approximately two-thirds women), one field supervisor from ISMS, a field coordinator, and a team leader (a medical doctor with a PhD or master’s degree). Two 3-day training classes were organized by ISMS staff with support from A&T, which built interview skills as well as provided insight on A&T, training on the survey questionnaire, and practical experience in using the questionnaire (mock interview and practice in the field). Standardized procedures for data collection, supervision, and coordination were also introduced and used consistently during data collection.

On average, each interview took 45 min to complete. At the end of each interview, we gave VND 40,000 (equal to USD 1.9) to each mother to compensate for travel expenses and time. The non-response rate was about 2%.

#### Variables

##### Outcome variables

Breastfeeding practices were assessed using four indicators recommended by the World Health Organization: 1) early initiation of breastfeeding, defined as the proportion of children born in the last 24 months who were put to the breast within 1 h of birth; 2) exclusive breastfeeding under 6 months, defined as the proportion of infants aged 0–5 months who were fed exclusively with breastmilk in the previous 24 h, with no foods, no liquids, and no water, with the exception of medications such as drops and syrups; 3) predominant breastfeeding under 6 months, defined as the proportion of infants aged 0–5 months who were fed breastmilk predominantly in the previous 24 h, with no foods, no energy-containing liquids such as non-human milk or food based liquids, but water, fruit juice, and medications such as drops, syrups are allowed; and 4) not using bottle and teats [21]. In addition, we also assessed four indicators for complementary feeding based on recommended practices and food consumed by the child in the previous 24 h: 1) timely initiation of complementary feeding, 2) minimum meal frequency, 3) minimum dietary diversity, and 4) minimum acceptable diet [21].

##### Exposure to the intervention

We chose women living in villages with an IYCF support group to represent exposure to the intervention. Although not all women in the villages who were selected for the evaluation participated in the support group in the previous 3 months, most IYCF practices, except for exclusive breastfeeding, were similar between those who attended and those who did not attend the IYCF support group in the previous 3 months **(**Additional file 3**)**.

##### Potential confounders

Maternal characteristics such as age, ethnicity (Kinh – a major ethnicity vs. other minority ethnicities), education (with > 9 vs. ≤ 9 years), and occupation (being a farmer vs. other occupations) were collected. We asked mothers about the place (hospital vs. out of hospital, typically at a commune health station) and mode of birth (vaginal vs. cesarean section). During the data analysis, we combined the place and mode of birth into three mutually exclusive categories: vaginal births outside hospitals, vaginal births in hospitals, and cesarean births in hospitals. To evaluate breastfeeding counseling during pregnancy, we asked, “When you were pregnant with (NAME), did you receive any advice about breastfeeding from anyone?” If yes, we asked, “By whom?” Those who indicated they had received advice from health workers or nutrition collaborators were considered to have received standard breastfeeding counseling during pregnancy. To evaluate the exposure to breastfeeding counseling and support at birth, we asked, “In the first 3 days after you gave birth to (NAME), did anyone show you how to breastfeed?” If yes, we asked, “By whom?” Those who indicated they had received advice from health workers or nutrition collaborators was considered to have received standard breastfeeding support at birth. The prevalence of household food security was also calculated based on guidelines developed by FANTA and USAID [22].

#### Data management and statistical methods

Survey data were double entered into EpiData 3.1 (The EpiData Association, Odense, Denmark); inconsistent values were validated using original hard copy questionnaires. All analyses were performed using survey commands to account for clustering in Stata 13.1 (Stata Inc., TX, USA). First, a bivariate analysis was used to compare background information and the prevalence of recommended feeding practices in communes with and without IYCF support groups, using a *p* < 0.05 based on two-sided χ^2^ test as the criterion for a statistical difference. Second, we performed multiple logistic regression analysis to examine associations between communes with and without access to IYCF support groups and recommended IYCF practices and adjusted for potential confounders.

## Results

### Implementation and coverage of the IYCF support group model in the nine provinces

**Fig. 2:**
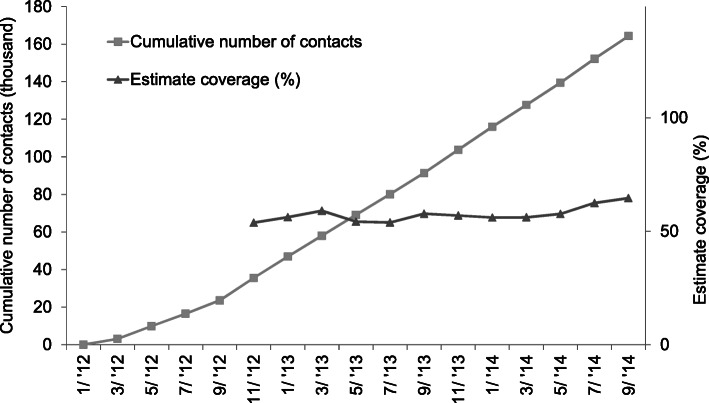
Cumulative number of contacts and coverage of the IYCF support group model (Alive & Thrive support group monitoring data from January 2012 to September 2014) in nine provinces

From November 2011 to November 2014, in partnership with the government, A&T supported the training of 1513 facilitators and establishment of 801 IYCF support groups in 267 villages across nine provinces. During 28 months of operation (which might have been shorter in provinces that launched the model later), facilitators contacted ~ 166,000 clients, including ~ 33,000 pregnant women and mothers with children aged 0–23 months in intervention villages in the IYCF support group meeting (Fig. 2). Between November 2012 to September 2014, the model covered about 60% of the total pregnant women or women with children aged 0–23 months in the villages with IYCF support groups (Fig. 2).

**Fig. 3:**
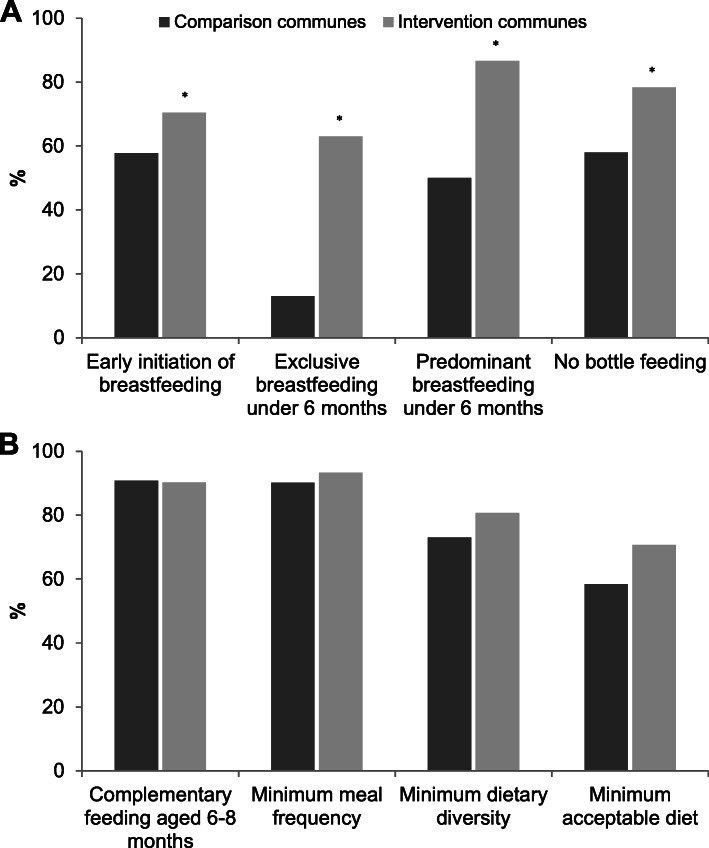
Select breastfeeding (**a**) and complementary feeding (**b**) practices by exposure to IYCF support group intervention (A&T IYCF support group assessment survey in 2014). Values are percentages, * differed from comparison communes; *p* < 0.05, two-sided χ^2^ test adjusting for clustering. We used data from mothers with children aged 0–23 months for early initiation of breastfeeding, no bottle feeding (*n =* 1110); aged 0–5 months for exclusive and predominant breastfeeding (*n =* 446); children aged 6–8 months (*n* = 137) for feeding of complementary feeding at 6–8 months; and children from aged 6–23 months (*n =* 664) for other three complementary feeding practices

### Costs for the IYCF support group model in the nine provinces

In total, A&T paid USD 496,500 for the model; of that, USD 186,000 was used for the village-level implementation, USD 210,000 was used to train village collaborators, and the remaining USD 100,500 was used for other costs at the central and provincial levels (Table 3). The expenditure for the implementation at the village level every month was USD 12.5 to support the meeting organization, USD 11 to compensate village collaborators (typically split into two person) and USD 5 for supportive supervision provided by staff from commune health stations (Table 3).

**Table 3:** Summary of the cost of the IYCF support group model covered by A&T

	Unit cost (USD)	No. of units	Unit type	Total per month (USD)	28 months of operation (USD)
Delivery costs					
Support the meeting organization	12.5	267	Villages per month	3198	89,554
Compensate village collaborators	11	267	Villages per month	3070	85,972
Supportive supervision by staff from commune health stations	5	78	Communes per month	374	10,465
Subtotal					185,991
Training cost					
Per diem (7 days training)	34	1513	Trainees		50,747
Transportation/logging	101	1513	Trainees		152,242
Lecturers (a class of 30)	134	50	Training		6766
Sub total					209,756
Other cost at the central and provincial levels					100,752
Total					496,500

Notes: There were five meetings every 2 months (Group 1 met once a month; Group 2 met once a month; and Group 3 met once every 2 months). The cost for organizing each meeting was about USD 5 (e.g., to buy water, fruits, and other snacks). The average cost for delivery of services were USD 15 per client and USD 3 per contact (inclusive of all expenditures) and were USD 5 and USD 1 (based on village-level implementation expenditures) over the 28 months of operation

### Effectiveness of the IYCF support group model

Survey participants in intervention and comparison communes had similar maternal, child, and household characteristics, with the exception of household food security levels that tended to be higher in intervention than comparison communes (61.5% vs. 46.5%, *p* = 0.06; Table 4**)**. In binary analysis, the prevalence of recommended breastfeeding practices was statistically higher (*p* < 0.05) in the intervention communes than that in the comparison communes: early initiation of breastfeeding (70.6% vs. 58.3%), exclusive breastfeeding under 6 months (62.8% vs. 13.1%), predominant breastfeeding under 6 months (86.6% vs. 49.8%), and no bottle feeding (78.0% vs. 60.0%) (Fig. 3). Compared with those in comparison communes, mothers in the intervention communes more often fed feed their children minimum dietary diversity (81.0% vs. 73.0%; *p* = 0.09) and minimum acceptable diet (70.6% vs. 58.3%; *p* = 0.06) (Fig. 3).

**Table 4:** Key maternal and child characteristics as well as the access to breastfeeding counseling and support (% or mean) by study round and A&T intervention area

	Comparison communes (*n* = 559)	Intervention communes (*n* = 551)
Household food secured	46.5	61.5
Mother characteristics:		
Age (y)	28.1	27.6
Kinh ethnicity	78.4	80.2
With ≥9 y of education	24.7	30.7
Being a farmer	48.3	53.7
The modes of births:		
Vaginal births outside hospitals	31.0	27.4
Vaginal births in hospitals	49.7	54.1
Cesarean births in hospitals	19.3	18.5
Child characteristics:		
Male	56.2	55.5
Age (months)	10.1	10.3

^*^ Differed from comparison communes (*p <* 0.05; χ^2^ test; using survey commands in Stata to account for clustering)

Logistic regression analysis controlling for potential confounding variables showed a higher prevalence of early initiation of breastfeeding (OR: 1.72; 95% CI: 1.08, 2.74), exclusive breastfeeding (OR: 12.52; 95% CI: 6.69, 23.44), predominant breastfeeding (OR 9.64; 95% CI: 5.07, 18.33), and no bottle feeding (with teats) (OR: 2.69; 95% CI: 1.82, 3.99) in communes with IYCF support groups compared to those without (Table 5). Timely initiation of complementary feeding, minimum meal frequency, minimum dietary diversity, and minimum acceptable diet tended to be higher among women with local access to IYCF support groups (Table 5). In addition, the prevalence of early initiation of breastfeeding was lower in vaginal births in hospitals (OR: 0.58; 95% CI: 0.37, 0.90) and cesarean births in hospitals (OR: 0.03; 95% CI: 0.02, 0.06) than vaginal birth outside of hospitals (data not shown). The prevalence of minimum dietary diversity was higher among mothers from the Kinh ethnicity compared to other ethnicities (OR: 2.55; 95% CI: 1.30, 4.99), mothers with ≥9 y of education compared with those with < 9 y (OR: 2.46; 95% CI:1.21, 5.01), and mothers in food-secure households compared with those in food-insecure households (OR: 1.69; 95% CI: 1.05, 2.73) (Table 5).

**Table 5:** Association (adjusted OR and 95% CI) between living in a community with an IYCF support group and breastfeeding and complementary feeding practices in mothers with children aged 0–23 months^a^

	Early initiation of breastfeeding^b^ (*n =* 1110)	Exclusive breastfeeding < 6 months (*n =* 446)	Predominant breastfeeding < 6 months (*n =* 446)	No bottle feeding (*n =* 1110)	Complementary feeding aged 6–8 months (*n =* 137)	Minimum meal frequency (*n =* 664)	Minimum dietary diversity (*n =* 664)	Minimum acceptable diet (*n =* 664)
Living in communes with an IYCF support group (ref. communes without an IYCF support group)	1.72* (1.08,2.74)	12.52*** (6.69,23.44)	9.64*** (5.07,18.33)	2.69*** (1.82,3.99)	1.03 (0.30,3.55)	1.22 (0.65,2.30)	1.33 (0.82,2.16)	1.51 (0.98,2.33)
Mother characteristics:								
Age (y)	1.01 (0.99,1.04)	1.02 (0.99,1.05)	0.99 (0.96,1.02)	1.01 (0.98,1.03)	1.00 (0.91,1.11)	0.95** (0.91,0.99)	1.00 (0.97,1.04)	1.01 (0.98,1.04)
Kinh ethnicity (vs. other ethnicities)	1.39 (0.90,2.16)	1.00 (0.57,1.74)	1.46 (0.69,3.10)	0.78 (0.51,1.21)	4.61 (0.91,23.26)	1.71 (0.74,3.93)	2.55** (1.30,4.99)	1.44 (0.87,2.40)
With ≥9 y of education (vs. < 9 y)	0.87 (0.62,1.23)	1.47 (0.85,2.56)	0.54 (0.27,1.07)	0.95 (0.72,1.26)	2.71 (0.61,12.09)	3.73** (1.46,9.51)	2.46* (1.21,5.01)	2.05** (1.25,3.39)
Being a farmer (vs. other occupations)	1.17 (0.78,1.75)	1.06 (0.61,1.84)	0.84 (0.48,1.45)	1.33 (0.93,1.91)	2.95 (0.56,15.37)	1.04 (0.52,2.09)	0.91 (0.57,1.43)	1.24 (0.76,2.02)
Household food secured (vs. unsecured)	1.07 (0.74,1.54)	0.93 (0.44,2.00)	1.29 (0.80,2.09)	0.94 (0.68,1.29)	0.78 (0.28,2.17)	1.66 (0.93,2.93)	1.69* (1.05,2.73)	1.4 (0.98,1.98)
Child characteristics:								
Male (vs. female)	1.13 (0.84,1.53)	1.01 (0.69,1.49)	0.85 (0.59,1.22)	0.96 (0.72,1.26)	3.84* (1.02,14.43)	1.56 (0.85,2.85)	0.84 (0.55,1.27)	1.05 (0.73,1.49)
Age (mo)		0.82* (0.70,0.97)	0.59*** (0.48,0.73)	0.98* (0.96,1.00)	4.40*** (1.94,9.96)	1.17** (1.05,1.30)	1.24*** (1.17,1.31)	1.07*** (1.03,1.11)

^a^Data from A&T IYCF support group assessment survey in 2014. Values are adjusted odds ratios (OR) and 95% CIs, using survey commands to account for clustering. Significantly different from the null value (OR = 1; two-sided *t* tests): * *p <* 0.05, ** *p <* 0.01, *** *p <* 0.001. ^b^Model also controlled the mode of birth and breastfeeding counseling during pregnancy and at birth)

## Discussion

The IYCF support group model reached the targeted populations residing in remote areas and plausibly contributed to improved early and exclusive breastfeeding practices [23]. The IYCF support groups improved breastfeeding practices more than complementary feeding practices.

First, the IYCF support group model could be implemented at scale to reach populations residing in remote areas. For less than USD 500,000, the model was scaled up in more than 267 remote villages across nine provinces and provided ~ 166,000 meeting/support contacts to ~ 33,000 pregnant women and mothers with children aged 0–23 months in 28 months of operation. At an average cost of just USD 15, the model could reach a woman an average of 5 times from the third trimester of pregnancy to 24 months after birth in remote, hard-to-reach areas in Vietnam. In Nigeria, the cost to reach one woman was USD 13 including training costs and USD 8.5 excluding training costs [8].

The IYCF support group model brought quality information and support to the women where they lived using credible facilitators in the villages [14]. Training traditional birth attendants helped to improve IYCF practices in Bangladesh [24] and peer counselling by mother support groups helped to increased early, exclusive, and continued breastfeeding in India [25]. In Nigeria, the implementation of the UNICEF’s Community IYCF Counselling Package helped to improved IYCF knowledge and counseling skills of health workers and community volunteers as well as improved IYCF knowledge and practices of mothers compared with baseline [8]. In our IYCF support group model, two groups, breastfeeding and complementary feeding, provided tailored information to women. Involvement of fathers and other family members in our model could have helped to change social norms and enhance support for mothers as shown in other studies [14, 26].

The targeting of women living in remote areas and belonging to ethnic minority groups is important. Previous studies in Vietnam showed a low prevalence of recommended IYCF practices among women belonging to ethnic minority groups [27] and varied across ethnic groups [28]. Among various reasons, lack of knowledge and skills and non-supportive social norms could inhibit adoption of recommended feeding practices [29]. Changing knowledge, skills, and norms can be challenging for women in mountainous areas due to lack of access to health information and support through health facilities (e.g., commune health stations, district health centers) or media (e.g., books, magazines, newspapers, and online materials) [30, 31]. Recently, the Government of Vietnam issued a Decision on the Implementation of National Target Program on Socio-economic Development of Remote Areas and among Ethnic Minority people for 2021–2030 that includes various programs on nutrition and health [32].

Second, the model was integrated into the existing health care system, thus maximizing the use of A&T resources, and improving sustainability [33]. The local village health or nutrition collaborators who coordinated the IYCF support group typically had some prior knowledge and skills relating to maternal, infant, and young child nutrition as well as communication and meeting facilitation skills [14]. With additional training and coaching supported by A&T and Departments of Health, the facilitators’ knowledge, skills, and materials were standardized [14]. The collaborators were typically considered credible people in the community, sensitive to cultural norms, and fluent in local dialects, allowing them to better facilitate the meeting [14]. In addition to the allowance from A&T, the facilitators may have received small allowances from other sources (e.g., the population, nutrition, health, and women unions’ collaborators), supplementing their income and motivating higher contributions to their villages’ activities. The facilitators could have integrated IYCF support group meetings with other content, such as content from farmers’ and women’s unions, which enhances participation [14]. The provincial and district trainers and supervisors received salaries from the government [14]. During the direct investment phase (2011–2014), most of the payments for trainers and supervisors were for travel and lodging. After that, the training and supportive supervision were less intensive and integrated with other trainings or supervision visits. By minimizing needs from external support and partner engagement, the model became sustainable beyond the project with funding from local government and other non-governmental organizations such as Save the Children and World Vision in Vietnam.

Third, we found that IYCF support group model has plausibly contributed to improved IYCF practices, especially exclusive and early initiation of breastfeeding. Previous studies showed water and premature feeding of complementary foods were key barriers towards exclusive breastfeeding [28]. With support from the facilitators, other women, husbands, and family members, the women could feed their babies only breastmilk. Previous studies revealed that interpersonal counseling and support can significantly improve the prevalence of exclusive breastfeeding [34]. A recent systematic review of 58 studies showed that of the WHO/UNICEF Baby-Friendly Hospital Initiative’s (BFHI) Ten Steps to Successful Breastfeeding, Step 10 – Foster the establishment of breastfeeding support groups (i.e., home peer counseling and support) and refer mothers to them on discharge from the hospital or clinic– is needed to sustain the benefit of the other nine steps on exclusive breastfeeding under 6 months and for continued breastfeeding [35]. The IYCF support group also helped to improve early initiation of breastfeeding, similarly to findings from to other studies in South Asia [24, 25]. Facilitators provided information and skills on breastfeeding after birth to women and their families, strengthening knowledge, beliefs, social norms, and self-efficacy to support the initiation of early breastfeeding. A&T also has interventions to improve breastfeeding and counseling and support through the Little Sun social franchise model in districts and provinces with IYCF support groups. Consistent messages and support from the villages, commune health stations, and district and provincial hospitals would help women to adopt the recommended breastfeeding practices.

The effect of the IYCF support group model weaker on early initiation of breastfeeding on exclusive breastfeeding. Mothers who gave birth in hospitals and via cesarean were less likely to initiate breastfeeding early. Going to the hospital (compared to commune health stations) to give birth could potentially affect the confidence women feel in making their own decision on in adopting recommended practices discussed within IYCF support groups. At the hospital, women might be more reliant on the decisions of health workers. In addition, cesarean births were a key barrier to early initiation of breastfeeding [36]. Suboptimal hospital protocols, inadequate training for staff, and facility routines for breastfeeding support may also play a role. At the time of the survey (early 2014), national guidelines for early essential newborn care (the First Embrace) in vaginal and cesarean births were not available in Vietnam [37], and early essential newborn care was only implemented in a few hospitals [37]. Lacking knowledge and skills relating to early essential newborn care, health workers may not have effectively supported the early initiation of breastfeeding. Early essential newborn care is only a part of the BFHI Ten Steps to Successful Breastfeeding [35]. A recent systematic review showed a positive association between the number of the BFHI steps the women were exposed to and increased prevalence of early and exclusive breastfeeding practices at hospital discharge, as well as any breastfeeding and exclusive breastfeeding duration [35]. In Vietnam, however, there were only 51 hospitals and maternities that had been accredited as BFHI back to the early 1990s and without follow-up reassessment [38]. The network of BFHI in Vietnam covered only 0.4% of the total hospitals and maternities; and subsequently, only 0.4% of births occurred in BFHI designated hospitals and maternities [38]. In Vietnam, increased coverage of BFHI designated hospitals and maternities is needed. In response to this need, the Vietnam decided to promote the implementing the Centers of Excellence for Breastfeeding model – the core components are the Ten Steps and the Code – by issuing Assessment Criteria and Designation Mechanism [39].

Fourth, complementary feeding practices tended to be better in mothers living in intervention than comparison communes. Previous studies suggested that a more intensive intervention, including enhanced interpersonal counseling, mass media campaigns, community mobilization, agriculture activities, and food supplementation would be needed to improve complementary feeding practices [8, 40–44]. In our study, the prevalence of minimum meal frequency was ~ 90% in both intervention and comparison communes, which indicates hunger might not be prevalent or a main driver of the low minimum acceptable diet prevalence. Dietary diversity was a challenge to achieve minimum acceptable diet. Households with food security, and mothers with at least 9 years of education, and belonging to the Kinh ethnicity, were more likely to achieve minimum acceptable diet. Women belonging to a higher socio-economic group might be able to purchase more diverse food items, and thus had a higher prevalence of minimum dietary diversity and acceptable diet. Similar results were found in other developing countries where poor access to recommended foods was one of the main barriers to recommended complementary feeding [8, 45]. To address this challenge, in addition to providing information and support for complementary feeding, interventions to help diversifying food production, food items available in the market, or increasing purchasing power of the family should be considered [46].

Our study has several strengths. In addition to findings on coverage, costs, and effectiveness, this is among the few studies that cover various aspects of an intervention model from the development, preparation, operation, and financial needs, which are typically in the gray literature. This study has limitations as well. The absence of baseline information and a randomized design altered our ability to consider possible pre-existing differences between the comparison and the intervention communes, which made the conclusions about effectiveness weaker than those from a randomized comparison trial. The budget for the assessment of the IYCF support group model only became available during the implementation of the IYCF model. Nonetheless, we had comparison communes to compare prevalence of the recommended practices. We did not have other verifications (e.g., observation of the IYCF support group meetings, in-depth interviews with mothers and facilitators, cross-checking lists of participants managed by facilitators) nor did we evaluate potential social desirability bias. Because participants in the communes with an IYCF support group could be more likely to say that they attended IYCF counseling and support or practiced recommended feeding practice than those in comparison communes, the true estimates of effectiveness could be smaller than the reported ones (i.e., be more toward the null hypothesis). For this manuscript, we were limited in estimating costs by only being able to include the costs based on money provided by A&T and no other costs, such as those incurred by the local government, providers, or clients [18]. Although the actual cost is unknown, we could propose a package that a donor may need which can be used in real settings when local governments typically contribute material, financial, and human resources to funded projects.

In conclusion, the IYCF support group model reached populations residing in remote areas and plausibly contributed to improved IYCF practices. Actions that were important for this model to reach remote populations were the integration with the existing health system, engagement with local partners from the early stages, contribution from local partners, maximum use of data to communicate about the outcomes and impact, and an exit strategy to advocate for the local government to sustain the model. This model can be scaled up in Vietnam or in similar settings. Research is needed on how best to undertake these actions to achieve sustainability given that donor funding, even if available, is almost always temporary.

## Supplementary Information

**Additional file 1.** An excel file with Monitoring and supportive supervision forms (in English and Vietnamese) used the IYCF support group.

**Additional file 2.** A PDF file with the Informed consent and Survey questionnaire.

**Additional file 3.** A PDF file with Sensitivity data analysis.

## Data Availability

Requests for data may be directed to the corresponding author and are subject to institutional data use agreements.

## Abbreviations

A&T: Alive & Thrive
IYCF: Infant and young child feeding
ISMS: Institute of Social and Medical Studies
USD: US dollar
VND: Vietnam Dong
BFHI: Baby Friendly Hospital Initiative
