# Supplementary material for: Community support model on breastfeeding and complementary feeding practices in remote areas in Vietnam: implementation, cost, and effectiveness
Source: Int J Equity Health. 2021 May 17;20:121. doi: 10.1186/s12939-021-01451-0 (PMC8127246; doi:10.1186/s12939-021-01451-0)
Supplement: Supplementary file 2 — Additional file 2. A PDF file with the Informed consent and Survey questionnaire. [file 12939_2021_1451_MOESM2_ESM.pdf]

ID | | | | | | | | | | | |

INFORMED CONSENT FORM
A&T Endline Survey of Mothers with a child under 24 months

Principal Investigators:
Nemat Hajeebhoy – Program Director, Alive & Thrive in Viet Nam
Nguyen Truong Nam –Director, Institute of Social and Medical Studies

Introduction/Purpose of research
We would like to invite you to participate in a study of mothers who have a child under 24 months of age.
The purpose is to evaluate the work of a program called "Alive & Thrive", which aims to improve the feeding of young children.

Research Procedure
If you agree to participate, the interviewer will ask you some questions about your knowledge, belief and practices on IYCF. The interview will take about 45 minutes.

Confidentiality
All information will be kept confidential and will be used only for the research purpose. Your personal information will be coded and kept confidential and then it will be destroyed as data is inputted and analyzed.

Risks
There will be no risks to you or your child's health when participating in this study.
Benefits
Your answers will benefit the community and country by helping the Alive & Thrive project make their programs for mothers and children more effective.
Incentives
After completing the interview, we would like to give you VND 40,000 to thank you for your participation.

Voluntary participation and withdrawal from the research
Your participation in the interview is completely voluntary. You can refuse to participate in the interview or answer any questions at any time. Even after agreeing to participate in the study, you can withdraw at any time in case of any inconvenience. Your withdrawal from the research will not prevent you from receiving services at the commune health center.

Do you have any questions?
Contact information
If you have any further question about the research, please contact Dr. Nguyen Truong Nam – Principal Investigator or Associate. Prof. Pham Van Hoan, Chairman of IRB:

Dr. Nguyen Truong Nam
Principle Investigator- Director of Institute of Social and Medical Studies
Address: No 50, Lane 141, Nguyen Khang Street, Yen Hoa Ward, Cau Giay District, Hanoi
Tel: 04.3555.8288/Fax: 04.3555.8274.

Dr. Pham Van Hoan
Chairman of IRB – Institute of Social and Medical Studies
Address: No 50, Lane 141, Nguyen Khang Street, Yen Hoa Ward, Cau Giay District, Hanoi
Tel: 04.3555.8288/Fax: 04.3555.8274.

Participant's commitment:
I am volunteering to participate in the research. I know that I can withdraw from the research at any time and the interviewer will answer any questions I may have.

____________________ ______________________________
DD/MM/YY Name of participant

Investigator's commitment:
I have explained the procedures involved in this research as well as the risks and benefits when participating in the research for voluntary participants.

__________________________ ______________________________
DD/MM/YY Name of data collector

A&T Endline Survey 2014
MOTHER OF A CHILD UNDER 24 MONTHS

Questionnaire ID ☐☐☐☐☐☐☐☐☐☐☐

1.* (Background and characteristics)** ***START TIME:______hour_______minute

No	Question	Code	
1.1	Field Supervisor's name/ code	……………………………………..	☐☐
1.2	Interviewer's name/ code	....	☐☐
1.3	Date of interview:	____/ _____ /2014	
1.4b	Province/ city that the interview is being conducted in:	Ha Noi 01; Hai Phong 31; Quang Binh 44; Quang Tri 45; Da Nang 48; Quang Nam 49; Khanh Hoa 56; Dak Lak 66; Dak Nong 67; Tien Giang 82; Ca Mau 96	
1.5b	District:	……………………………………………..	☐☐
1.6	Commune:	....	☐☐☐
1.7	Village/ hamlet:	....	☐☐
1.8	What is your name/code of mother?	....	☐☐
1.9	What is your ethnicity?	Kinh .... 1<br>Other (specify) 7	
1.10	What is your birth date?<br>***(Remind respondent to use the solar calendar. If she does not remember her birth date, ask mother's age.)***	Birth date: ____/ _____ /______<br>OR<br>Age: ☐☐ years old	
1.11	What is your marital status?<br><br>***Use show card.***	Unmarried .... 1<br>Married .... 2<br>Widowed/Divorced/Separated .... 3<br>Other (specify)...7<br>...	
1.12b	What level of education have you **<u>completed</u>**?	Never attended schools ....0<br>≤ 5 years ....1<br>6-9 years ....2<br>10-12 years ....3<br>> 12 years 4	
1.13	How many children from 2 to 5 years do you have?<br>***Include adopted or fostered children if respondent is primary care-giver.***	☐ children	
1.14	How many children under 24 months do you have?	☐ infants	
1.15	Name of the index child (the youngest child under 24 months old)	....	
1.16	Is (NAME) a boy or a girl?	Boy..1<br>Girl..0	
1.17	What is (NAME's) birth date?<br>***Remind the mother to use the solar calendar.***	____/ _____ /______<br>The baby is:<br>1.................. <6 months<br>2. .... 6-23.9 months	

1.18	Mother's main occupation before delivery?	Farmer (planting, feeding animals, farming)/fisherman .... 1<br>Salary government employee .... 2<br>Salary non-government employee (including factory worker) .... 3<br>Small trader/ self-employment /self owned business/services (tailor/hairdresser/builder)/freelancer .... 4<br>Housewife/unemployment/university student/pupil .... 5<br>Other (specify) 7	
1.18b	Before giving birth to (NAME), did you contribute to the Social Security Fund?<br>*This is the money taken from your paycheck. Then the fund provides some money to you during sickness, maternity, labor accident, old age…*	Yes ....1<br>No 0	
1.19b	Have you gone back to work (e.g., to the field, previous employment)?	Yes ....1<br>No 0	**0→1.27**
1.20b	How long after giving birth to (NAME) did you go back to work?	……….. month(s)……….. days	
1.22	In the past week, how many days did you work outside the home?	☐ Days	
1.23	On days you work outside the home, for how many hours are you away on average?	☐☐ Hours	
1.21	Do you take (NAME) with you when you work outside the home?	Yes .... 1<br>No 0	
1.27	Who does (NAME) live in the same house with for at least 15 days in the last 30 days?<br><br>***Multiple responses possible.***<br>***Probe:*** Anyone else?	With me (the mother)................................1<br>My husband (the father/step father).............2<br>Siblings ....3<br>Grandparents...4<br>Other relative ....5<br>Nanny (hired child care-giver).....................6<br>Other (specify)..7	
1.28	Who looks after (NAME) on a daily basis?<br><br>***Multiple responses possible.***<br>***Probe:*** Anyone else?	Me (the mother) ....1<br>My husband (the father/step father).............2<br>Siblings ....3<br>Grandparents...4<br>Other relative ....5<br>Nanny (hired child care-giver).....................6<br>Teachers (in kind garden) ....8<br>Other (specify)..7	

2. (Breastfeeding Practice)

Now I would like to ask you some questions about pregnancy, delivery and breastfeeding.

No	Question	Code	
2.2b	When you were **pregnant** with (NAME), did you receive any advice about breastfeeding from **anyone**?	Yes .... 1<br>No 0	**0→2.3c**
2.3b	**From whom** did you receive this advice?<br><br>***Multiple responses possible***<br>***Probe:*** Anyone else?	Husband... 0<br>Mother/Mother in law............................. 1<br>Other Family members .... 2<br>Neighbors/ Friends/co-workers .... 3<br>Hamlet health worker/nutrition collaborator .... 4<br>Women Union staff .... 5<br>Midwife/nurse .... 6<br>Doctor/physician assistant .... 7<br>Other (specify) 8	

2.3c	When you were **pregnant** with (NAME), did you receive any advice about breastfeeding from **any other sources**?<br><br>***Multiple responses possible***	None .... 0<br>Yes, books/newspapers/magazines.......... 1<br>Yes, television .... 2<br>Yes, internet (computer, phone…) .... 3<br>Yes, loudspeaker/radio.......... 5<br>Yes, other events.......... 4	
2.4b	Where did you give birth to (NAME)?	Hospital (including private & public, regional clinic, district health center)...... 1<br>Commune health center .... 2<br>Other health facility .... 3<br>At home.......... 4<br>Other (specify) 7	**2→2.6**<br><br>**4→2.12**
2.5	Did you have a cesarean section when you gave birth to (NAME)?	Yes .... 1<br>No 0	**1→2.7**
2.6	Did you have an episiotomy when you gave birth to (NAME)? (An episiotomy is when, during delivery, the vagina is cut to help the infant come out).	Yes .... 1<br>No 0	
2.7	For how many days after you gave birth to (NAME) did you stay in the health facility?	[ ][ ] Days	
2.9	Did you or your family member bring any infant formula to health facility when you went to give birth to (NAME)?	Yes .... 1<br>No 0	
2.10	Did you or your family member purchase any infant formula at or near the health facility shortly after you gave birth to (NAME)?	Yes .... 1<br>No 0	
2.11	Were you given any **FREE** infant formula once in the health facility?	Yes .... 1<br>No 0	
2.12	Did (NAME) ever breastfeed?<br><br>***(The infant was breastfed if he/she ingested any breast milk. Include feeding mother's milk by spoon, cup or bottle or from another mother.)***	Yes .... 1<br>No 0	**0→3.1b**
2.14	How soon after birth did you put (NAME) to the breast for the first time?<br><br>**If the mother answers "immediately", interviewers ask the mother again about the exact time and record the appropriate time.**<br>***If less than 1 hour, circle 1.***<br>***If less than 24 hours, circle 2 and record hours.***<br>***If more than 24 hours, circle 3 and record days.***	Within 1 hour .... 1<br>Number of hours .... 2<br>Number of days .... 3<br><br>[ ][ ] . [ ] Hours [ ][ ] Days	
2.15b	Some mothers give things other than breast milk to the newborn right after birth. Thinking about the first 3 days after birth, was (NAME) given any …<br><br>***READ EACH RESPONSE (1-5).***<br>**Note: first 3 days after birth**<br>***Multiple responses possible***	Plain water .... 1<br>Sugar or glucose water .... 2<br>Honey .... 3<br>Infant Formula /other infant milk.......... 4<br>Lemon juice/ herbal tea (eg licorice root).5<br>Anything else? (specify) .... 6<br>[Gave nothing besides breast milk].......... 7	
2.16	Did you give (NAME) the first milk (colostrum)?<br><br>*Colostrum is breast milk was produced in 1-3 first days after birth*	Yes .... 1<br>No 0	
2.18	**In the first 3 days** after you gave birth to (NAME), did anyone show you how to breastfeed?	Yes .... 1<br>No 0	**0→2.20**

2.19	Who showed you how to breastfeed?<br><br>***Multiple responses possible.***<br>***Probe:*** Anyone else?	Husband.. 0<br>Mother/Mother in law............................ 1<br>Other Family members .... 2<br>Neighbors/ Friends/co-workers .... 3<br>Hamlet health worker/nutrition collaborator .... 4<br>Women Union staff .... 5<br>Midwife/nurse .... 6<br>Doctor/physician assistant .... 7<br>Other (specify) .... 8<br>...	
2.20	Are you still breastfeeding (NAME)?	Yes ....1<br>No 0	**1⮊2.25**
2.21	How old was (NAME) when you stopped breastfeeding?<br>**(*If answer is not numeric, probe for approximate number*)**	………. month(s)……….. days<br><br>Never breastfed ……………………….. 0	
2.22b	Why did you stop breastfeeding?<br><br>***Multiple responses possible.***<br><br>***Probe***: Anything else?	Mother sick .... 0<br>Problem with breast (pain, cracked nipples, engorgement) .... 1<br>Not enough time to feed........................... 2<br>Mother felt not enough breastmilk .... 3<br>Mother felt her breast milk not good ....10<br>Mother got pregnant/new infant born .... 4<br>Mother went back to work .... 5<br><br>Infant refuses/lazy to eat .... 9<br>Infant resisted breastfeeding (due to sick or natural)... 6<br>Infant already grown up .... 7<br>Other (specify) 8	
2.25	Did you **ever** have a **problem** with breastfeeding (NAME)?	Yes ....1<br>No...0	**0⮊ Module 3.1**
2.26	What kind of problem?<br><br>***Multiple responses possible.***<br><br>***Probe:*** Anything else?	Infant resists to breastfeed ....10<br>Infant did not suck well/did not attach well .... 4<br>Infant was choking, vomiting, refluxing ...11<br>Infant was sick, teething or got thrush......12<br>Infant did not grow as expected.................13<br>I don't have enough milk (says/thinks) .... 1<br>Problems with breast (pain, crack nipples, engorgement) .... 2<br>Pain associated with episiotomy or C-Section…………………………………...3<br>Not enough time to breastfeed, have to go to work .... 5<br>I was sick and unable to breastfeed...........6<br>I did not know how to and was not confident with breastfeeding.....................................8<br>My parents or husband urged me to give infant formula or other foods to my kids ...9<br>Other (Specify)..7	
2.27	Did you seek help or advice from anyone for the problem?	Yes ....1<br>No………………………………………0	**0⮊2.30**
2.28	From whom **did you get support** to address these **difficulties**?	Mother/Mother in law............................ 1<br>Husband .... 2<br>Other Family members 3	

	Multiple responses possible.<br><br>***Probe:*** Anyone else?	Neighbors/ Friends/co-workers................ 4<br>Hamlet health worker/nutrition collaborator ....5<br>Women Union staff.................................6<br>Midwife/nurse .... 7<br>Doctor/physician assistant .... 8<br>Other (specify) .... 9<br>...	
2.30	**In the last 3 months**, did you have any difficulties in breastfeeding (NAME)?	No breastfeeding in the last 3 months........2<br>No difficulty..0<br>Yes 1	**0, 2 ➩ Module 3.1**
2.31	What type of the breastfeeding difficulties did you experience in the last 3 months?<br><br>***Multiple responses possible.***<br><br>***Probe:*** Anything else?	Infant resists to breastfeed ....10<br>Infant did not suck well/did not attach well .... 4<br>Infant was choking, vomiting, refluxing ...11<br>Infant was sick, teething or got thrush ......12<br>Infant did not grow as expected ....13<br>I don't have enough milk (says/thinks) .... 1<br>Problems with breast (pain, crack nipples, engorgement) .... 2<br>Pain associated with episiotomy or C-Section……………………………...3<br>Not enough time to breastfeed, have to go to work .... 5<br>I was sick and unable to breastfeed............6<br>I did not know how to and was not confident with breastfeeding.....................................8<br>My parents or husband urged me to give infant formula or other foods to my kids ...9<br>Other (Specify)..7	
2.32	Did you seek help or advice from anyone for the problem?	Yes ....1<br>No………………………………………0	**0➩ Module 3.1**
2.33	From whom **did you get support** to address these **breastfeeding difficulties**?<br><br>***Multiple responses possible***<br>***Probe:*** Anyone else?	Mother/Mother in law............................. 1<br>Husband ....2<br>Other Family members ....3<br>Neighbors/ Friends/co-workers............... 4<br>Hamlet health worker/nutrition collaborator ....5<br>Women Union staff................................6<br>Midwife/nurse .... 7<br>Doctor/physician assistant .... 8<br>Other (specify) 9	

3.1. (Feeding Practices)

Now I would like to ask you some questions about how (NAME) was fed.

2.23	How many times did you breastfeed (NAME) yesterday from the time you (***the mother***) got up to the time you went to bed?<br>***(If answer is not numeric, probe for approximate number)***	Number of day time feedings:<br>☐☐<br>*Check question 2.20, if the baby was stopped breastfeeding, write 00*	
2.24	How many times did you breastfeed (NAME) last night from the time you (***the mother***) went to bed until you got up?<br>***(If answer is not numeric, probe for approximate number)***	Number of night time feedings:<br>☐<br>*Check question 2.20, if the baby was stopped breastfeeding, write 00*	

			Yes	No. of times (Ask only for white cells)	No	DK
3.1b	Thinking about **the time period from when (NAME) woke up yesterday morning until the time s/he woke up this morning**, was (NAME) given any . . . ***(READ LIST)*** *. . . ?*	2. Plain water	1		2	8
		3. Infant formula	1		2	8
		4. Other milk (e.g. Packaged milk, Fresh milk, Condensed milk)	1		2	8
		6. Packaged fruit juice/ sugar water/herbal tea	1		2	8
		7. Clear broth/rice water/soup	1		2	8
		8. Other fluids (e.g. pepsi, coca…)	1		2	8
		12. Vitamins, minerals, syrup drop	1		2	8

3.1.3	Thinking about all day yesterday and last night, was (NAME) given any water to clean out his/her mouth.	Yes ....1<br>No ....0<br>Don't know/don't remember 8	
3.1.1	**From the time (NAME) woke up yesterday morning until time s/he woke up this morning**, did (NAME) drink anything from a bottle with a nipple?	Yes ....1<br>No 0	**1⮚Module 3.2**
3.1.5	Do you have a bottle with a nipple for [NAME]?	Yes ....1<br>No 0	

3.2.(Foods) We have talked about breastfeeding and we have talked about liquids that you fed (NAME) yesterday. Now I would like to ask you about any **OTHER** foods (NAME) eats.

No.	Question	Code	
3.2.1	Does (NAME) eat any semi-solid foods?	Yes.................... 1<br>No 0	**0⮚4**
3.2.2	*Main meal: Rice/ porridge/ flour/ noodle…*<br><br>Thinking about **the time period from when (NAME) woke up yesterday morning until the time s/he woke up this morning**,<br>how many times did (NAME) eat a main meal?	Number of meals: ☐	
3.2.3	What is the average meal size (NAME) ate?<br><br>***Show the mother a common, 200ml bowl. If bowl she uses is larger or smaller, describe size.***	☐☐ ☐ bowl(s)<br>Describe the size of the bowl	
3.2.4	*Snacks: biscuits, fruit, snacks, yogurt...*<br><br>Think about **the time period from when (NAME) woke up yesterday morning until the time s/he woke up this morning**, how many times did s/he eat a snack? Do not count small feeds such as one or two bites of your food.<br>***Formula and other liquids mentioned in 3.1b should NOT be counted as a snack.***	Number of snacks: ☐	

Thinking about **the time period from when (NAME) woke up yesterday morning until the time s/he woke up this morning**, was (NAME) given any. . . . *READ RESPONSES* . . . ?

No	Question	Code		
3.2.5		Yes	No	DK
	a. Rice/Rice porridge/ rice flour	1	0	8
	b. Noodles (rice or instant or green bean noodle)	1	0	8
	c. Pumpkins, carrots, squash or sweet potatoes that are yellow or orange inside	1	0	8
	d. White potatoes, white yams, cassava, or any other foods made from roots	1	0	8

	e. Ripe mangoes, papaya, water melon, tomatoes, tangerine	1	0	8
	f. Any dark green, leafy vegetables	1	0	8
	g. Any other fruits or vegetables	1	0	8
	h. Eggs (chicken or ducks or geese)	1	0	8
	i. Liver, kidney, heart or other organ meats	1	0	8
	j. Any meat, such as beef, pork, lamb, goat, chicken, or duck	1	0	8
	k. Fish, prawn, crabs, shellfish, eel, snails	1	0	8
	l. Fish sauce	1	0	8
	m. Tofu or any food make from soy bean	1	0	8
	n. Any other beans, peas, lentils, or nuts	1	0	8
	o. Cheese, yogurt, condensed milk, flan/pudding or other milk products	1	0	8
	p. Any oil, fats, or butter with food	1	0	8
	q. Any sugary foods such as chocolates, sweets, candies, pastries, cakes, or biscuits	1	0	8
	s. Baby cereal (e.g., Heinz, Hipp, Cerelac / Nestle, Ridielac / Vinamilk, Gerber)	1	0	8
	r. Any other liquid or food item, not mentioned above<br>***If Yes, specify* ....**	1	0	8

3.3.Difficulties in Feeding Semi-Solid and Solid Foods

No	Question	Code	
3.3.1	Did you face any concerns or difficulties when you started feeding semi-solid foods to (NAME)?	Yes .... 1<br>No 0	**0⮱3.3.5**
3.3.2	What concerns or difficulties?<br><br>***Probe***: Any others?<br><br>***Multiple responses possible.***	Infant refusal or infant spits it out, choking, vomiting, refluxing... 1<br>Infant was sick, teething or got thrush........................ 2<br>Infant had a poor appetite/infant was lazy to eat .... 3<br>Infant got diarrhea, constipation, allergy, after eating .... 8<br>Infant did not grow as expected................................. 9<br>I does not have time to prepare/feed, has to go to work .....<br>Family does not have resources................................. 5<br>I did not know how to and was not confident with preparing food for the baby .... 10<br>Family members discouraged certain foods/practices.. 6<br>My parents or husband did not support me consistently.. 11<br>Lack of people who can help me .... 12<br>Other (specify).. 7	
3.3.3	Did you seek help or advice from anyone for the concerns or difficulties?	Yes……………………………………………1<br>No…………………………………………..............0	**0⮱3.3.5**
3.3.4	From whom **did you get support** to address these **feeding difficulties**?<br><br>***Multiple responses possible***<br>***Probe:*** Anyone else?	Mother/Mother in law... 1<br>Husband..2<br>Other Family members ....3<br>Neighbors/ Friends/co-workers .... 4<br>Hamlet health worker/nutrition collaborator ………...5<br>Women Union staff..6<br>Midwife/nurse .... 7<br>Doctor/physician assistant .... 8<br>Other (specify) 9	
3.3.5	In the last 3 months, Did you face any concerns or difficulties when you feeding semi-solid foods to (NAME)?	Yes .... 1<br>No ……………………………………………...0	**0⮱Module 4**

3.3.6	What are the difficulties?<br><br>***Multiple responses possible.***	Infant refusal or infant spits it out, choking, vomiting, refluxing<br>.. 1<br>Infant was sick, teething or got thrush....................... 2<br>Infant has a poor appetite/infant was lazy to eat......... 3<br>Infant got diarrhea, constipation, allergy, after eating<br>..8<br>Infant did not grow as expected................................9<br>Mother does not have time to prepare/feed, has to go to work .... 4<br>Family does not have resources................................. 5<br>I did not know how to and was not confident with preparing food for the baby ....10<br>Family members discouraged certain foods/practices...6<br>My parents or husband did not support me consistently...11<br>Lack of people who can help me ....12<br>Other (specify).. 7	
3.3.7	Did you receive any help or advice from anyone for the concerns or difficulties?	Yes..1<br>No……………………………………………0	**0→Module 4**
3.3.8	From whom **did you get support** to address these **feeding difficulties**?<br><br>***Multiple responses possible***<br>***Probe:*** Anyone else?	Mother/Mother in law.. 1<br>Husband.. 2<br>Other Family members .... 3<br>Neighbors/ Friends/co-workers .... 4<br>Hamlet health worker/nutrition collaborator ………….5<br>Women Union staff.. 6<br>Midwife/nurse .... 7<br>Doctor/physician .... 8<br>Other (specify) .... 9<br>...	

4. **(Illness and feeding during illness)** Now I would like to ask you about any illness (NAME) had in the past two weeks.

No	Question	Code	
4.1	Was (NAME) ill in the past two weeks?	Yes .... 1<br>No 0	**0→Module 6.0**
4.2	If yes, what symptoms were his/her main symptoms?<br><br>***Multiple responses possible.***	Fever...1<br>Cough/Cold ....2<br>Fast breathing/shortness of breath.........................3<br>Diarrhea...4<br>Other (specify) 7	
4.3	When (NAME) had these symptoms, did you breastfeed (NAME) . . . ?<br><br>***Use show card***	Much less than usual ....1<br>A little bit less than usual ....2<br>About the same as usual.......................................3<br>More than usual..4<br>Not at all..5<br>Not currently breastfeeding.................................6	
4.4	When (NAME) had these symptoms, did you give (NAME) . . .?<br>***Use show card***	Much less to eat than usual ....1<br>A little bit less to eat than usual ....2<br>About the same to eat as usual.............................3<br>More to eat than usual..4<br>Stopped giving food entirely................................5<br><br>Does not feed semi-solid and solid foods yet........6	

6 **(Determinants)**

6.0 (Intention)

Now I want you to think ahead to a time when you might have another child. For these next statements, please respond for the actions you would take if you had another child. Please tell me whether you strongly disagree, disagree, disagree somewhat, agree somewhat, agree or strongly agree with each of the following statements. **[*As you read the responses, point to each box*.]** Please put your finger on the box to indicate how strongly you disagree or agree with each of the following statements.

If the answer is "don't know," code as 8.

1	2	3	4	5	6
**Strongly disagree**	**Disagree**	**Somewhat Disagree**	**Somewhat Agree**	**Agree**	**Strongly agree**

No	Question	Code
6.0.7	If I have another child, I will breastfeed him within 1 hour after giving birth	
6.0.2	If I have another child, I will not give him anything other than breast milk in the first 3 days after birth.	
6.0.1	If I have another child, I will not give him any water to drink or to wash out his mouth for the first 6 months.	
6.0.3	If I have another child, I will not give him any infant formula for the first 6 months.	
6.0.4	If I have another child, I will not give him any liquids besides breast milk for the first 6 months.	
6.0.5	If I have another child, I will not give him any semi-solid or solid foods for the first 6 months.	
6.0.6	If I have another child, I will not give him any food, water, or infant formula for the first 6 months.	
6.0.8	If I have 6 months paid maternity leave or have sufficient financial support from family, I will feed my infant **ONLY** breast milk and **NO** other food, water or infant formula, until s/he completes 6 months.	
Check	*Interviewer check Question No 1.17 and* ***circle appropriate number:***<br>1. *Child < 6 months → Only ask Question No 6.0.9 and 6.0.10*<br>2. *Child 6-23 months → Only ask Question No 6.0.11 and 6.0.12*	
6.0.9	*Ask mother with children < 6 months:* I will go back to work only when my child is 6 months or older.	
6.0.10	*Ask mother with children < 6 months:* when my child reaches 6 months of age, I will feed him iron-rich foods such as red meat, eggs and leafy green vegetables.	
6.0.11	*Ask mother with children 6-23 months:* If I have another child, I will take 6 months maternity leave.	
6.0.12	*Ask mother with children 6-23 months:* If I have another child, I will feed him iron-rich foods such as red meat, eggs and leafy green vegetables when he/she reaches 6 months of age.	

***6.1.* (Knowledge of breastfeeding)** Now we would like to ask your opinion about infant feeding.

No	Question	Code	
6.1.1b	How long after birth should a newborn start breastfeeding?<br><br>**If the mother answers "immediately", interviewers ask the mother again about the exact time and record the appropriate time.**<br>***If less than 1 hour, circle 1.***<br>***If less than 24 hours, circle 2 and record hours.***<br>***If more than 24 hours, circle 3 and record days.***	Within 1 hour .... 1<br>Number of hours .... 2<br>Number of days .... 3<br>[ ][ ] Hours [ ][ ] Days<br><br>Don't know 8	

6.1.4b	If a mother thinks her 4-month-old infant is not getting enough breast milk, what should she do?<br><br>***Multiple responses possible.***<br><br>***Probe once:*** Anything else?	Breastfeed more often/more frequently .... 0<br>Give infant formula .... 1<br>Give other liquids/foods (e.g. water/fruits juice /Rice porridge/ rice flour).. 2<br><br>Mother needs to drink more water .... 3<br>Mother needs to eat more food .... 4<br>Mother needs to eat special food .... 5<br><br>Refer to health care workers (doctor, nurse, midwife, nutrition collaborator/village health worker) .... 6<br>Other (Specify): .... 7<br>Don't know 8	
6.1.5	In the figure, which picture shows correct attachment, 1 or 2?<br>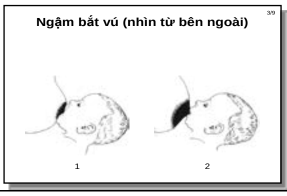 	Position 1 is correct...1<br>Position 2 is correct...2<br>Both of these positions is correct ....3<br>Neither of these positions is correct ....4<br>Don't know 8	
6.1.6	Each time you breastfeed, do you think you should give a little from each breast or empty one breast before switching to the other?	A little from each breast .... 1<br>Empty one breast before switching to the other .... 2<br>Don't know 8	
6.1.7	Which is better for an infant under 6 months, breast milk alone or a combination of breast milk and infant formula?	Breast milk alone...1<br>A combination of breast milk and infant formula ....2<br>Don't know 8	
6.1.9	Until what month should a mother give her infant **ONLY** breast milk and **NO** other foods, water or infant formula?	☐☐ Months<br><br>Don't know…………………………………..…...........98<br>No response……………………………….. 99	
6.1.10	In what month do you think an infant should **START** receiving plain water in addition to breast milk?	☐☐ Months<br>From birth ....0<br>Don't know………………………………….............98<br>No response..99	
6.1.11	In what month do you think an infant should first **START** to receive liquids other than water in addition to breast milk?	☐☐ Months<br>From birth…..0<br>Don't know ....98<br>No response..99	
6.1.12b	Until what month should a mother continue to breastfeeding?<br><br>***Write down the age as months.***	☐☐ Months<br>Don't know ....98<br>No response..99	

6.2. (Knowledge related to feeding semi-solid and solid foods)

Now we would like to ask your opinions about feeding semi-solid and solid foods

No	Question	Code
6.2.1	After completing what month should an infant first start to receive semi-solid foods (e.g. cereal, rice flour) in addition to breast milk?	☐☐ Months<br>Don't know 98

<table>
<tr><td rowspan="3">6.2.2</td><td rowspan="3">How many meals per day should a <u>breastfed infant</u> at following ages eat?

(Ask for each age group and each meal/snack)
Main meal: Rice/Rice porridge/ rice flour/noodle
Snack: bisquit, fruit, bimbim, yogourt…</td><td>6-8 months</td><td>9-11 months</td><td>12-23 months</td></tr>
<tr><td>Main meal ☐

Don't know………9.8</td><td>Main meal ☐

Don't know…………98</td><td>Main meal ☐

Don't know…………98</td></tr>
<tr><td>Snacks ☐

Don't know……….98</td><td>Snacks ☐

Don't know…………98</td><td>Snacks ☐

Don't know…………98</td></tr>
<tr><td>6.2.3</td><td>On average, how much food should an infant 6-8 months old be offered each time you feed him?

Show respondent a picture of the commonly used bowl (200 ml). If different, ask respondent to describe the size of the bowl.</td><td colspan="3">1/3 bowl 1
1/2 bowl 2
2/3 bowl 3
1 bowl 4
More than 1 bowl 5
Don't know .... 8</td></tr>
</table>

<table>
<tr><td rowspan="12">6.2.4</td><td colspan="3">According to you, at which month of age should you <u>START</u> giving an infant the following foods?</td></tr>
<tr><td>Bread</td><td>☐☐</td><td rowspan="11">Less than 1 month (0-29 days)...0
1 month (30-59 days)................ 1
2 months (60-89 days) 2
3 months 3
4 months 4
5 months 5
6 months 6
7 months 7
8 months 8
9 months 9
10 months 10
11 months 11
12 months 12
After 12 months 13
Don't know ....98</td></tr>
<tr><td>Rice</td><td>☐☐</td></tr>
<tr><td>Vegetables</td><td>☐☐</td></tr>
<tr><td>Fruits</td><td>☐☐</td></tr>
<tr><td>Meat</td><td>☐☐</td></tr>
<tr><td>Organ meats (liver, kidney, heart, etc.)</td><td>☐☐</td></tr>
<tr><td>Fish (all types), shrimp, crab....................................</td><td>☐☐</td></tr>
<tr><td>Eggs (chicken, duck, goose, etc.)</td><td>☐☐</td></tr>
<tr><td>Peanuts, other nuts</td><td>☐☐</td></tr>
<tr><td>Infant formula milk</td><td>☐☐</td></tr>
<tr><td>Milk (all other kinds of milk excluding breast milk)</td><td>☐☐</td></tr>
</table>

<table>
<tr><td>6.2.6</td><td>What are some foods that are rich in iron?

Multiple responses possible.

Do NOT read responses.</td><td>Red meat (e.g., pork, beef).......... 1
Organ meats / blood (e.g., liver, kidneys, heart).......... 2
Egg yolks 3
Fish, shrimp, crab 0
Green vegetables (e.g. katuk , amaranth, watercress, morning glory) 4
Fortified food (e.g. infant cereal, formula milk).......... 5
Supplementation vitamin/mineral (including syrup and medicine) 6
Other (specify).......... 7
Don't know/don't know about iron foods .... 8</td></tr>
</table>

6.3. (Beliefs)

I would like to ask your opinion about some other feeding practices. Please tell me whether you strongly disagree, disagree, disagree somewhat, agree somewhat, agree or strongly agree with each of the following statements. ***As you read the responses, point to each box.*** Please put your finger on the box to indicate how strongly you disagree or agree with each of the following statements.

If the answer is "don't know," code as 8.

1	2	3	4	5	6
**Strongly disagree**	**Disagree**	**Somewhat Disagree**	**Somewhat Agree**	**Agree**	**Strongly agree**

No	**Question**	**Code**
	Please tell me your opinion about the following statements.<br>***"I" in all of the following statements refers to the respondent and not the interviewer.***	
6.3.26	If I breastfeed my infant within 1 hour after giving birth, It'll be good for my child's health	
6.3.27	If I breastfeed my infant within 1 hour after giving birth, It'll be good for my health	
6.3.2	If I am breastfeeding, but **DO NOT** give my newborn infant formula during the first 24 hours after birth, s/he will be hungry.	
6.3.1	If I am breastfeeding, but **DO NOT** give my infant water until s/he completes 6 months, my infant will be thirsty.	
6.3.4	If I feed my infant **ONLY** breast milk and **NO** other food, water or infant formula, until s/he completes 6 months, I am giving my infant all the nutrients s/he needs to be healthy.	
6.3.6	If I continue to breastfeed my infant when s/he has diarrhea, it could make the diarrhea worse.	
6.3.7	If I feed my infant a combination of breast milk and infant formula until s/he completes 6 months, I am giving him/her the **BEST** possible nutrition.	
6.3.8	If **DO NOT** clean my infant's mouth out with water after breastfeeding, my infant will get thrush.	
6.3.10	If I am breastfeeding my 5 month old infant, but **DO NOT** give my infant water, s/he will be too hot.	
6.3.3	If I express my breast milk and store it in the refrigerator for less than 3 days, this milk is still good for my infant.	
6.3.17	After I start to give infant formula to my infant, my body will produce less breastmilk.	
6.3.11	If I am breastfeeding and I wait until my infant has completed 6 months old to start feeding her/him semi-solid or solid foods, it is good for my infant's health.	
6.3.12	If I feed my infant a combination of breast milk and other foods when s/he is between 4 and 6 months of age, I am giving my infant the best possible nutrition.	
6.3.13	If a woman has small breasts, she will have difficulty producing enough breast milk to feed her infant.	
6.3.16	If I continue breastfeeding until my infant completes two years, it is good for my infant's health.	
6.3.18	If I feed my infant **ONLY** breast milk and **NO** other food, water, or infant formula until he completes 6 months, I am giving my infant all the nutrients s/he needs for optimal brain development.	
6.3.19	If I give my infant organ meats like heart, liver, and kidney, starting at 6-8 months, it is good for his/her health.	
6.3.24	A mother who returns to work when her infant is 4 months old will have to use mainly formula to feed her infant.	
6.3.25	If I feed my child iron-rich foods starting at 7 months, it will help with brain development	

6.4 (Social Norms)

I would like to ask your opinion about some social norms of other feeding practices. Please tell me whether you **strongly disagree, disagree, disagree somewhat, agree somewhat, agree or strongly agree** with each of the following statements. ***As you read the responses, point to each box.*** Please put your finger on the box to indicate how strongly you disagree or agree with each of the following statements.

If the answer is "don't know," code as 8.

1	2	3	4	5	6
**Strongly disagree**	**Disagree**	**Somewhat Disagree**	**Somewhat Agree**	**Agree**	**Strongly agree**

No	Question	Code
6.4.14	**Most people who are important to me (e.g. family members, friends…)** think that a mother after normal delivery, can breastfeed her infant within 1 hour.	
6.4.15	**Most people who are important to me (e.g. family members, friends…)** think that a mother after caesarean section, can breastfeed her infant within 1 hour.	
6.4.16	**Most people who are important to me (e.g. family members, friends…)** think that infant needs feeding formula milk in the first week after birth.	
6.4.1	**Most people who are important to me (e.g. family members, friends…)** think that I should feed my infant only breast milk, and no other food, water, or infant formula for the first 6 months.	
6.4.3	Most people who are important to me (e.g. family members, friends…) approve of me giving my baby water before she/he reaches 6 months of age.	
6.4.4	Most people who are important to me (e.g. family members, friends…) approve of me giving my baby infant formula before she/he reaches 6 months of age.	
6.4.5	Most people who are important to me (e.g. family members, friends…) approve of me giving my baby semi-solid or solid foods before s/he reaches 6 months of age.	
6.4.24	Most people who are important to me (e.g. family members, friends…) approve of me giving my baby organ meats like heart, liver and kidney between 6-8 months of age.	
6.4.10	Most people who are important to me think that a big child is healthy	
6.4.20	Most people who are important to me (e.g. family members, friends…) think that I should breastfeed my infant for at least 12 months	
6.4.21	Most people who are important to me (e.g. family members, friends…) approve of me using a bottle with a nipple to feed my infant milk, water, juice…	
6.4.17	**Most women who have infants like me** feed their infant breast milk within 1 hour after normal delivery.	
6.4.18	**Most women who have infants like me** feed their infant breast milk within 1 hour after caesarean section.	
6.4.19	Most people who are important to me feed their infant formula milk in 1 first week.	
6.4.2	**Most women who have infants like me** feed their infant only breast milk, and no other food, water, or infant formula for the first 6 months.	
6.4.6	Most mothers who have infants like me give their babies water before they reach 6 months of age.	
6.4.7	Most mothers who have infants like me give their babies infant formula before they reach 6 months of age.	
6.4.8	Most mothers who have infants like me give their babies semi-solid or solid foods before they reach 6 months of age.	
6.4.3_bl	Most women who are like me give organ meats like heart, liver and kidney to their infants between 6-8 months of age.	
6.4.12	Most mothers who have infants like me think that a big child is healthy	
6.4.22	Most mothers who have infants like me breastfeed their babies for at least 12 months	

6.4.23	Most mothers who have infants like me use a bottle with a nipple to feed their children milk, water, juice…	

6.5 (Self-Efficacy)

I would like to ask your opinion about some other feeding practices. Please tell me whether you are **very unconfident, unconfident, somewhat unconfident, somewhat confident, confident, or very confident** in response to the statement. ***As you read the responses, point to each box.***

If the respondent does not know, code 8.

1	2	3	4	5	6
**Very unconfident**	**Unconfident**	**Somewhat unconfident**	**Somewhat confident**	**Confident**	**Very confident**

No	**Question**	**Code**
6.5.1	My body can produce enough colostrum to feed my newborn within one hour after birth.	
6.5.3	My body can produce enough breast milk to feed my newborn only breast milk and no water or infant formula in the first 24 hours.	
6.5.4	The “first milk” produced by my body is all my newborn needs in the first 24 hours after birth.	
6.5.5	My breast milk is of good enough quality to nourish my infant so that the infant does not need any other food, water, or infant formula until s/he has completed 6 months.	
6.5.6	If I go back to work before my infant is six months old, I will have to start feeding him infant formula or semi-solid/solid foods.	
6.5.7	The more I breastfeed my infant, the more breast milk my body will produce.	
6.5.8	If I need good advice from a doctor about breastfeeding, I am able to get it.	
6.5.9	If my mother-in-law wants to feed my newborn infant formula in the first 24 hours after birth, I can refuse to let her do it.	
6.5.10	I know where to find a doctor I trust to get good advice about starting to feed my infant semi-solid and solid foods.	
6.5.15	I can continue to breastfeed my infant at least in 12 months	
6.5.11	I can continue to breastfeed my infant until he reaches 24 months of age.	
6.5.12	I can feed my infant organ meats like heart, liver, and kidney starting at 6-8 months.	
6.5.13	I can **refrain** from giving my infant water before s/he reaches 6 months of age.	
6.5.14	I can convince other caretakers of my infant to **not** give him/her water to drink before s/he reaches 6 months of age.	

7. (Utilization)

7.1. (Exposure to Franchise) Now I would like to ask you some questions about where you might have gotten information about infant feeding.

**No**	**Question**	**Code**	
7.1.1	Have you ever seen the logo “Mặt trời bé thơ” before?<br><br>***Showcard***	Yes ....1<br>No..............................0	
7.1.2	Have you ever heard the name “Mặt trời bé thơ” before?	Yes...1<br>No 0	**0→Module 7.2.b**
7.1.3	Do you know anyone who has ever been to “Mặt trời bé thơ” counseling service?	Yes...1<br>No 0	**0→7.1.5**
7.1.4	How many women have you spoken who have been to “Mặt trời bé thơ”?	☐ women	

7.1.5	Did you receive an invitation card to go to "Mặt trời bé thơ"?	Yes..1<br>No 0	
7.1.6	Have you ever been to "Mặt trời bé thơ" counseling service?	Yes..1<br>No 0	**0➾Module 7.2.b**
7.1.7	How many times have you been to this service?	☐ Times	
7.1.9b	How many times did you go to "Mặt trời bé thơ" when you/(NAME) was. . . ***Read responses*** . . . ?	(see table below)	
7.1.26	Have you ever received "Mặt trời bé thơ" counseling services along with others such as other mothers, husband, your parents? Who are they?<br>***Multiple responses possible.***	No, just by myself ....1<br>Yes, with other mothers................................2<br>Yes, with my husband ....3<br>Yes, with my parents 4	
7.1.27	What, **specifically**, did they advise you to do?<br><br>***Probe:*** Anything else?<br><br>***If response is general, probe for a more specific response.***<br><br>***Multiple responses possible.***	About nutrition for pregnant and lactating women . 0<br>Putting infant to breast immediately after birth....... 1<br>Giving only colostrum in the first day or two until breastmilk comes in .... 2<br>No pre-lacteals in the first 3 days .... 3<br>Nurse more leads to more breast milk.................. 13<br>How to breastfeed well(position to breastfeed, empty one breast before switching to the other…) 16<br>Feed only breast milk up to six months .... 4<br>No water in first 6 months.................................... 14<br>Feeding mashed food after 6 months...................... 5<br>Ensure that complementary foods include all needed components (starch, protein, vitamin & mineral, iron, lipids).. 15<br>Washing hands with water and soap before preparing/feeding infant .... 8<br>Feeding the infant an extra meal or extra food after illness .... 9<br>Give infant formula .... 10<br>Mother and child not in diet .... 11<br>Bring child to " Mat troi be tho" room................. 12<br>Other (Specify) .... 97<br>Don't know/don't remember 98	
7.1.19 b	How **attractive** is the facility of "Mặt trời bé thơ" ?	(see scale below)	
7.1.21 b	How **useful** are the advice from"Mặt trời bé thơ"?	(see scale below)	
7.1.24	Will you return to "Mặt trời bé thơ" in the future?	Yes... 1<br>No 0	

7.1.9b:

	Times
Pregnant	
Delivering	
0 - 5 months	
Only ask mothers with children 6-23 months<br>6 - 23 months	

7.1.19 b:

1	2	3	4	5	6
Very un-attractive	Un-attractive	Somewhat un-attractive	Somewhat attractive	Attractive	Very attractive

7.1.21 b:

1	2	3	4	5	6
Very useless	Useless	Somewhat useless	Some what useful	Useful	Ver□ useful

7.2.b (Exposure to IYCF support group)

No	Question	Code	
7.2.7	In the past 3 months, have you participated in a community **IYCF support group**?	Yes.. 1<br>No 0	**0➾Module 7.2**

7.2.8	How many sessions have you attended?	☐☐ sessions	
7.2.9	What, **specifically**, did they advise you to do?<br><br>***Probe:*** Anything else?<br><br>***If response is general, probe for a more specific response.***<br><br>***Multiple responses possible.***	About nutrition for pregnant and lactating women . 0<br>Putting infant to breast immediately after birth....... 1<br>Giving only colostrum in the first day or two until breastmilk comes in .... 2<br>No pre-lacteals in the first 3 days .... 3<br>Nurse more leads to more breast milk.................. 13<br>How to breastfeed well(position to breastfeed, empty one breast before switching to the other…)........... 16<br>Feed only breast milk up to six months .... 4<br>No water in first 6 months.................................... 14<br>Feeding mashed food after 6 months....................... 5<br>Ensure that complementary foods include all needed components (starch, protein, vitamin & mineral, lipids)... 15<br>Washing hands with water and soap before preparing/feeding infant .... 8<br>Feeding the infant an extra meal or extra food after illness .... 9<br>Give infant formula .... 10<br>Mother and child not in diet .... 11<br>Bring child to " Mat troi be tho" room................. 12<br>Other (Specify) .... 97<br>Don't know/don't remember 98	
7.2.10	How **friendly the collaborator is? *(use showcard)***	1 Very unfriendly<br>2 Unfriendly<br>3 Somewhat unfriendly<br>4 Somewhat friendly<br>5 Friendly<br>6 Very friendly	
7.2.11	How **useful** is the advice /support from the collaborator to improve your feeding practice? ***(use showcard)***	1 Very useless<br>2 Useless<br>3 Somewhat useless<br>4 Some what useful<br>5 Useful<br>6 Very useful	
7.2.12	How **useful is** the support from other mothers to improve your feeding practice**? *(use showcard)***	1 Very useless<br>2 Useless<br>3 Somewhat useless<br>4 Some what useful<br>5 Useful<br>6 Very useful	
7.2.13	How **useful is** the meeting with you in other aspects such as networking, learning experience in improving household income? ***(use showcard)***	1 Very useless<br>2 Useless<br>3 Somewhat useless<br>4 Some what useful<br>5 Useful<br>6 Very useful	
7.2.14	Will you return to "**IYCF support group**" in the future?	Yes...1<br>No...0	

7.2 (Exposure to other health providers)

Now I would like to ask you some questions about exposure to health providers, other than at "Mặt trời bé thơ"

No	Question	Code	
7.2.1	In the past 3 months, has a doctor or nurse in a health facility ***[besides at "Mặt trời bé thơ"]*** given you advice about feeding (NAME)?	Yes .... 1<br>No... 0	**0→7.2.3**
7.2.2	What, **specifically**, did they advise you to do?<br><br>***Multiple responses possible.***<br><br>***Probe:*** Anything else?<br><br>***If response is general, probe for a more***	About nutrition for pregnant and lactating women..0<br>Putting infant to breast immediately after birth ....1<br>Giving only colostrum in the first day or two until breastmilk comes in .... 2<br>No pre-lacteals in the first 3 days .... 3<br>Nurse more leads to more breast milk ....13<br>How to breastfeed well(possition to breastfeed, empty one breast before switching to the other…) 16	

	specific response.	Feed only breast milk up to six months .... 4<br>No water in first 6 months ....14<br>Feeding mashed food after 6 months ....5<br>Ensure that complementary foods include all needed components (starch, protein, vitamin & mineral, lipids) .... 15<br>Washing hands with water and soap before preparing/feeding infant .... 8<br>Feeding the infant an extra meal or extra food after illness .... 9<br>Give infant formula..10<br>Mother and child not in diet..................................11<br>Bring child to " Mat troi be tho" room...................12<br>Other (Specify) .... 97<br>Don't know/don't remember 98	
7.2.3	In the past 3 months, has a village health worker or nutrition collaborator given you advice about feeding (NAME)?	Yes .... 1<br>No.. 0	**0⮊7.2.5**
7.2.4	What, **specifically**, did they advise you to do?<br>***Multiple responses possible.***<br><br>***Probe once:*** Anything else?<br><br>***If response is general, probe for a more specific response.***	About nutrition for pregnant and lactating women..0<br>Putting infant to breast immediately after birth ....1<br>Giving only colostrum in the first day or two until breastmilk comes in .... 2<br>No pre-lacteals in the first 3 days .... 3<br>Nurse more leads to more breast milk ....13<br>How to breastfeed well(possition to breastfeed, empty one breast before switching to the other…) 16<br>Feed only breast milk up to six months .... 4<br>No water in first 6 months ....14<br>Feeding mashed food after 6 months ....5<br>Ensure that complementary foods include all needed components (starch, protein, vitamin & mineral, lipids) .... 15<br>Washing hands with water and soap before preparing/feeding infant .... 8<br>Feeding the infant an extra meal or extra food after illness .... 9<br>Give infant formula..10<br>Mother and child not in diet..................................11<br>Bring child to " Mat troi be tho" room..................12<br>Other (Specify) .... 97<br>Don't know/don't remember 98	
7.2.5	In the past 3 months, have you attended a meeting/workshop where breastfeeding was the topic of discussion?	Yes .... 1<br>No.. 0	**0⮊ Module 8**
7.2.6	How many times did you attend a meeting/workshop?	☐☐ times	

8. (Media exposure)

Now I would like to ask you some questions about media exposure

No	Question	Code	
8.1	Do you ever watch TV?	Yes...1<br>No 0	**0⮊8.16**
8.2b	How often do you watch TV?<br><br>***showcard***	Daily (7 days/week) .... 1<br>Several times a week (2-6 days/week) .... 2<br>About once a week .... 3<br>Less than once a week (≤ 3 days/month).................... 4	

		Don't know/don't remember.................................... 8	

8.3b	What are the 2 TV channels you usually watch?<br><br>***Multiple responses possible.***	**National Channels:**<br>VTV1 11 | VTV3 13 | Other national Channels 18<br>**Channel of Province:**<br>Ha Noi 1 | Quang Tri 45 | Dak Lak 66<br>Thai Nguyen 19 | Da Nang 48 | Dak Nong 67<br>Hai Phong 31 | Quang Nam 49 | Tien Giang 82<br>Thanh Hoa 38 | Quang Ngai 51 | Vinh Long 86<br>Quang Binh 44 | Khanh Hoa 56 | Ca Mau 96<br><br>**Other TV: ....**98 Specify 1-2 channels: ………………………

8.4b	What kinds of TV programs do you watch most often?<br><br>***Multiple responses possible.***<br>***Probe:*** Anything else?<br><br>***If mother responds "entertainment," probe:*** What kind of entertainment?	News.. 1<br>Music.. 2<br>Children's program/cartoons.................................... 3<br>Sports.. 4<br>Movie .... 5<br>Game shows .... 6<br>Health/disease programs .... 7<br>Cooking program... 8<br>Science/life/education programs .... 10<br>Agriculture program.. 11<br>Weather program.. 12<br>Other (specify)...98	
8.5	Generally, at what times do you watch TV?<br><br>***Multiple responses possible.***<br>***Probe:*** At any other time?<br>***If times mentioned overlap multiple categories, circle each category that applies.***	0:00 – < 6:00 ....1<br>6:00 – < 9:00 ....2<br>9:00 – < 12:00 ....3<br>12:00 – < 15:00 ....4<br>15:00 – < 18:00 ....5<br>18:00 – <21:00 ....6<br>21:00 – < 24:00 7	
8.6	During last 30 days, did you see any **advertisements about infant formula** on the television?	Yes ....1<br>No ....0<br>Don't know/don't remember......................................8	 **0⮩8.8**<br>**8⮩8.8**
8.7	In the past 30 days, about how often did you see an advertisement about **infant formula on the television**? Was it . . .<br>***showcard***	Daily (7 days/week) .... 1<br>Several times a week (2-6 days/week) .... 2<br>About once a week .... 3<br>Less than once a week (≤ 3 days/month)................... 4<br>Don't know/don't remember.................................... 8	
8.8	**<u>Now, I am no longer asking about formula advertisements</u>**. During last 30 days, did you see any information on **<u>breastfeeding</u>** on television?	Yes .... 1<br>No .... 0<br>Don't know/don't remember.................................... 8	 **0⮩8.16**<br>**8⮩8.16**
8.9	In the past 30 days, how often did you see information on **<u>breastfeeding</u>** on television? Was it .<br>***show card***	Daily (7 days/week) .... 1<br>Several times a week (2-6 days/week) .... 2<br>About once a week .... 3<br>Less than once a week (≤ 3 days/month)................... 4<br>Don't know/don't remember.................................... 8	

TVC2: NURSE MORE AND NO WATER TVCS (DO NOT READ TITLE)

8.16	***Show Picture set 2 and ask:***<br>Have you ever seen a video clip with these snapshots below?	Yes ....1<br>No ....0<br>Don't know/don't remember.................................... 8	 **0⮩8.26**<br>**8⮩8.26**

8.17	In which TV channels did you see the video clips?<br><br>***Multiple responses possible.***	**Never seen on the TV** .... 0<br><br>**National Channels:**<br>VTV1 11 | VTV3 13 | Other national Channels 18<br><br>**Channel of Province:**<br>Ha Noi 1 | Quang Tri 45 | Dak Lak 66<br>Thai Nguyen 19 | Da Nang 48 | Dak Nong 67<br>Hai Phong 31 | Quang Nam 49 | Tien Giang 82<br>Thanh Hoa 38 | Quang Ngai 51 | Vinh Long 86<br>Quang Binh 44 | Khanh Hoa 56 | Ca Mau 96<br><br>**Other TV:** 98 Specify 1-2 channels: ………………………<br>**Don't remember** 99

8.18	Where have you seen this video clip besides the TV?<br>***Multiple responses possible.***	Only on the TV .... 0<br>Mobile phone/computer/internet .... 1<br>TV Screen in health facility .... 2<br>TV Screen in supermaket .... 3<br>Other events e.g., seminar.. 4<br>Other (specify) ....6<br>...	
8.92	In the past 30 days, how often did you see this video clip?<br><br>***Use show card***	Daily (7 days/week) .... 1<br>Several times a week (2-6 days/week) .... 2<br>About once a week .... 3<br>Less than once a week (≤ 3 days/month) .... 4<br>Don't know/don't remember..................................... 8	
8.19	What are the key messages you could recall after watching the video clips?<br><br>***Multiple responses possible.***<br>***Probe:*** Anything else?	Nurse more leads to more breast milk.........................1<br>Breastfed -> Signal-> More breast milk......................2<br>Exclusive breastfeeding for children < 6 months ....3<br>Continue to breastfeed if you worry you don't have enough milk...4<br><br>Breast milk has enough water ....5<br>No water for children < 6 months ....6<br>No rinsing mouth with water for children < 6 months 7<br>A few drops of water can make your baby sick...........8<br><br>Breast milk has enough nutrients ....9<br>No formula for children < 6 months ....10<br><br>Breast milk makes baby smart..................................11<br>Breast milk makes baby healthy ....12<br>Leading organizations recommended breastfeeding in the first 6 months ....13<br>Other (specify)..……98<br>...	
8.20	What did you do after watching the video clips?<br><br>***Multiple responses possible.***<br>***Probe:*** Anything else?	Became more confident in breastfeeding the child ....1<br>Followed the recommendation from the TVC................2<br>Helped/supported others with child feeding practices ...3<br>Discussed the information with others ....4<br>Sought for additional breastfeeding information from health care providers, books, internet… ....5<br>Sought for additional breastfeeding information from relatives, neighbors, friends, co-workers… ....6<br>Did nothing 0	
8.26	***Show Picture set 2 and ask:***<br><br>Have you ever seen these images in other occasions, besides on video	No .... 0<br>Yes, posters.. 1<br>Yes, bill boards (out of home) .... 2<br>Yes, bus wrap 3	

	format?	Yes, books, magazine .... 4<br>Yes, leaflets .... 5<br>Yes, website, Facebook, FanPage .... 6<br>Yes, other events e.g., seminar 7	

Now, I am asking you about approach to the information on **selecting, preparing, and feeding of complementary foods** on television

8.31	During last 30 days, did you see any information on **selecting, preparing, and feeding of complementary foods** on television?	Yes .... 1<br>No .... 0<br>Don't know/don't remember 8	**0→8.36**<br>**8→8.36**
8.32	In the past 30 days, how often did you see information on **selecting, preparing, and feeding of complementary foods** on television? Was it . .<br><br>***Use Show card***	Daily (7 days/week).... 1<br>Several times a week (2-6 days/week) .... 2<br>About once a week .... 3<br>Less than once a week (≤ 3 days/month).... 4<br>Don't know/don't remember 8	

TVC3: THE IRON RICH FOOD TVC (DO NOT READ TITLE)

8.36	***Show Picture set 3 and ask:***<br>Have you ever seen a video clip with these snapshots below?	Yes .... 1<br>No .... 0<br>Don't know/don't remember 8	**0→8.46**<br>**8→8.46**
8.37	In which TV channels did you see the video clips?<br><br>***Multiple responses possible.***<br>***Probe:*** Anything else?	**Never seen on the TV** .... 0<br>**National Channels:**<br>VTV1 11 | VTV3 13 | Other national Channels 18<br>**Channel of Province:**<br>Ha Noi 1 | Quang Tri 45 | Dak Lak 66<br>Thai Nguyen 19 | Da Nang 48 | Dak Nong 67<br>Hai Phong 31 | Quang Nam 49 | Tien Giang 82<br>Thanh Hoa 38 | Quang Ngai 51 | Vinh Long 86<br>Quang Binh 44 | Khanh Hoa 56 | Ca Mau 96<br>**Other TV:** 98 Specify 1-2 channels: ……………………<br>**Don't remember** 99	
8.38	Where have you seen this video clip besides the TV?<br>***Multiple responses possible.***	Only on the TV.... 0<br>Mobile phone/computer/internet .... 1<br>TV Screen in health facility .... 2<br>TV Screen in supermaket .... 3<br>Other events e.g., seminar .... 4<br>Other (specify) 6	
8.93	In the past 30 days, how often did you see the video clip?<br><br>***Use show card***	Daily (7 days/week) .... 1<br>Several times a week (2-6 days/week) .... 2<br>About once a week .... 3<br>Less than once a week (≤ 3 days/month) .... 4<br>Don't know/don't remember.... 8	
8.39	What are the key messages you could recall after watching the video clips?<br><br>***Multiple responses possible.***<br>***Probe:*** Anything else?	Iron rich foods helps brain development.... 21<br>Iron rich foods prevent anemia.... 22<br>Iron is found in foods like liver, egg, red meat .... 23<br>Iron is found in green vegetables (e.g. katuk , amaranth, rau den, watercress, morning glory) .... 24<br>Leading health organizations recommend feeding iron rich foods.... 25<br>Start feeding iron rich foods from 6 months onwards.... 26<br>Other (specify) .... ……98<br>....	

8.40	What did you do after watching the video clips?<br><br>***Multiple responses possible.***<br>***Probe:*** Anything else?	Became more confident in feeding iron-rich foods for the child .. 1<br>Planned to give egg yolk when the child is at 6 mo-olds .... 2<br>Planned to give the child animal liver (e.g., pig, chicken, cow) when the child is at 6 mo-olds .... 7<br>Planned to give the child red meat (e.g., pork, beef) when the child is at 6 mo-olds .... 8<br>Planned to give the child green leafy vegetables (e.g. katuk , amaranth, rau den, watercress, morning glory)when the child is at 6 mo-olds .... 9<br>Helped/supported others with child feeding practices .... 3<br>Discussed the information with others .... 4<br>Sought for additional information about feeding iron-rich foods from health care providers, books, internet… .... 5<br>Sought for additional information about feeding iron-rich foods from relatives, neighbors, friends, co-workers… .... 6<br>Did nothing 0	
8.46	***Show Picture set 3 and ask:***<br><br>Have you ever seen these images in other occasions, besides on video format?	No...0<br>Yes, posters ....1<br>Yes, bill boards (out of home) ....2<br>Yes, bus wrap ....3<br>Yes, books, magazine...4<br>Yes, leaflets ....5<br>Yes, website, Facebook, Fan Page ....6<br>Yes, other events e.g., seminar...7	

Show Pictures and ask:

8.50	***Show to pictures set 4***<br><br>Have you ever been given or purchased any of the booklet?	Yes .... 1<br>No 0	
8.51	***Show to pictures set 5***<br><br>Have you ever been given or purchased any of the booklet?	Yes .... 1<br>No 0	
8.52	***Show to pictures set 6***<br><br>Have you ever been given any of these leaflets?	Yes .... 1<br>No 0	
8.53	***Show to pictures set 7***<br><br>Have you ever been given any of these leaflets?	Yes .... 1<br>No 0	
8.54	***Show to pictures set 8***<br><br>Have you ever been given any of these leaflets?	Yes .... 1<br>No 0	
8.60	**Now, I would like to ask you about information in loudspeaker** .<br>During last 30 days, did you listen to information about feeding from loudspeaker?	Yes .... 1<br>No 0	**0➾ Module 11**
8.61	In the past 30 days, how often did you listen to the information from loudspeaker? Was it . .<br><br>***Use show card***	Daily (7 days/week)... 1<br>Several times a week (2-6 days/week) .... 2<br>About once a week .... 3<br>Less than once a week (≤ 3 days/month)......................... 4<br>Don't know/don't remember 8	

8.62	What are the key messages you could recall after listening to loudspeaker?<br><br>***Multiple responses possible.***<br>***Probe:*** Anything else?	About nutrition for pregnant and lactating women............ 0<br>Putting infant to breast immediately after birth................. 1<br>Giving only colostrum in the first day or two until breastmilk comes in .... 2<br>No pre-lacteals in the first 3 days .... 3<br>Nurse more leads to more breast milk .... 13<br>How to breastfeed well(possition to breastfeed, empty one breast before switching to the other…)..................... 16<br>Feed only breast milk up to six months .... 4<br>No water in first 6 months .... 14<br>Feeding mashed food after 6 months............................... 5<br>Ensure that complementary foods include all needed components (starch, protein, vitamin & mineral, lipids) 15<br>Washing hands with water and soap before preparing/feeding infant .... 8<br>Feeding the infant an extra meal or extra food after illness .... 9<br>Give infant formula... 10<br>Mother and child not in diet.. 11<br>Bring child to " Mat troi be tho" room .... 12<br>Other (Specify) .... 97<br>Don't know/don't remember 98	

11. (Household economic status) We're almost finished. I would like to ask you some questions about the characteristics of your household.

If interviewing in the household, observe as much as possible. If interviewing outside the household, ask the following questions.

No	Question	Code	
11.1	Main materials of the house **floor**	Hard /pressed soil ground... 1<br>Crude bamboo wattle/ Rattan..................................... 2<br>Polished wooden .... 3<br>Rough brick, cement, brickbat .... 4<br>Ceramics, marble, enameled tiles................................ 5<br>Other (Specify)... 7	
11.2	Materials of the main **roof**	Leaflet, straw... 1<br>Bamboo, rattan, tree trunk... 2<br>Oil paper... 3<br>Configured zinc sheets .... 4<br>Wooden .... 5<br>Fibro cement.. 6<br>Tiles.. 7<br>Concrete .... 8<br>Other (Specify).. 9	
11.3	Materials of the main **wall**	No walls .... 1<br>Cane, tree trunk .... 2<br>Clay .... 3<br>Plywood, carton, recycle wrap .... 4<br>Concrete .... 5<br>Brick (different kinds)…………………………………………6<br>Shuttering .... 7<br>Other (specify) 8	
11.4	Does your house have a bathroom?	Yes .... 1<br>No... 0	

11.5	What kind of toilet does your family use?	No latrine (the discharge goes to river, sea, lake, garden...)……………………………………………0<br>Sharing with animals..1<br>One pit latrine..2<br>Two pit latrine(2-compartment latrine)........................3<br>Septic tank..4<br>Semi-septic tank..5<br>Hanging/ Into piped sewage system........................6<br>Other (specify) 7	
11.6	What is the **main source of water** that your family uses for drinking?	Rain ....1<br>Tap-water ....2<br>Pumped well-water..3<br>Well-water..4<br>Lake-Pool ....5<br>River, Stream..6<br>Bottled water ....7<br>Other (specify) 8	
11.7	What is the main **energy** source does your family usually use for cooking?<br><br>***(Disregard those used for livestock)***	Electricity ....1<br>Contained liquid gasoline..2<br>Biogas..3<br>Petroleum ....4<br>Peat, Charcoal, Coal..5<br>Firewood, Straw, Hay ....6<br>Other (specify) 7	
11.8	Does your family have an **electricity** system for use?	Yes ....1<br>No..0	
11.9	Does your family have any of the following things?<br><br>***Read responses.***<br><br>***Yes..........................1***<br>***No...........................0***	1. TV	☐
		2. Video player, DVD Player	☐
		3. Radio cassette/stereo player	☐
		4. Telephone	☐
		5. Cell/mobile phone	☐
		6. Computer	☐
		7. Refrigerator, ice cube ctorage	☐
		8. Air conditioners	☐
		9. Washing machine	☐
		10. Gas cooker	☐
		11. Electric cooker, rice cookers	☐
		12. Cooling and heating water machine	☐
		13. Mattress	☐
		14. Sofa	☐
		15. Wardrobes of various kind	☐
		16. Electric fan of various kind	☐
		17. Bicycle	☐
		18. Motorcycle	☐
		19. Boat	☐
		20. Car	☐
		21. Pumping machine	☐
		22. Rice milling machine	☐
		23. Feeding grinding machine	☐

		24. Grain harvesting machine	☐
		25. Tractor/tractor plough	☐
		26. Cart	☐
		27. Pig	☐
		28. Cow/buffalo/horse	☐
		29. Goat/sheep	☐
		30. Poultry (Chicken/duck/geese)	☐
11.10	Do you have a vegetable **garden** that supplies some of your food?	Yes .... 1<br>No........ 0	
11.11	Is there a **fish pool at your house that supplies some of your food?**	Yes .... 1<br>No........ 0	

12. (Household food security)

"Rarely" means 1-2 times in the past 30 days.
"Sometimes"means 3-10 times in the past 30 days.
"often" more than 10 times in the past 30 days.

No	Question	Code	
12.1	In the past 30 days did you worry that your household would not have enough food?	Yes .... 1<br>No 0	**0⮫12.3**
12.2	If "Yes", how often did this happen?	Rarely........ 1<br>Sometimes........ 2<br>Often 3	
12.3	In the past 30 days were you or any household members not able to eat the kinds of foods you preferred because you didn't have enough money to buy them?	Yes .... 1<br>No 0	**0⮫12.5**
12.4	If "Yes", how often did this happen?	Rarely........ 1<br>Sometimes........ 2<br>Often 3	
12.5	In the past 30 days did you or any household member eat just a few kinds of food day after day because you didn't have enough money to buy different foods?	Yes .... 1<br>No 0	**0⮫12.7**
12.6	If "Yes", how often did this happen?	Rarely........ 1<br>Sometimes........ 2<br>Often 3	
12.7	In the past 30 days, did you or any household member eat food that you did not want to eat because you didn't have enough money to obtain other types of food?	Yes .... 1<br>No 0	**0⮫12.9**
12.8	If "Yes", how often did this happen?	Rarely........ 1<br>Sometimes........ 2<br>Often 3	
12.9	In the past 30 days, did you or any household member eat a smaller meal than you felt you needed because there was not enough food?	Yes .... 1<br>No 0	**0⮫12.11**
12.10	If "Yes", how often did this happen?	Rarely........ 1<br>Sometimes........ 2<br>Often 3	
12.11	In the past 30 days did you or any household member eat fewer meals in a day because there was not enough food?	Yes .... 1<br>No 0	**0⮫12.13**
12.12	If "Yes", how often did this happen?	Rarely........ 1<br>Sometimes........ 2<br>Often 3	
12.13	In the past 30 days was there ever no food at all in your household because there were no money to get more?	Yes .... 1<br>No 0	**0⮫12.15**
12.14	If "Yes", how often did this happen?	Rarely........ 1<br>Sometimes........ 2	

		Often 3	
12.15	In the past 30 days, did you or any household member go to sleep at night hungry because there was not enough food?	Yes .... 1<br>No 0	0➩12.17
12.16	If "Yes", how often did this happen?	Rarely..................... 1<br>Sometimes............. 2<br>Often 3	
12.17	In the past 30 days, did you or any household member go a whole day without eating anything because there was not enough food?	Yes .... 1<br>No 0	0➩13.1
12.18	If "Yes", how often did this happen?	Rarely..................... 1<br>Sometimes............. 2<br>Often 3	

***Record end time.* ________________________**

13. Anthropometry

Weight when holding infant (kg) (time 1) (without shoes and robe)	☐☐ ☐ Kg
Weight when holding infant (kg) (time 1) (without shoes and robe)	☐☐ ☐ Kg

No	Indicators	First time	Second time
For mothers of infants aged 0-23.9 months			
13.1	Weight (kg)<br>(without shoes and robe)	☐☐, ☐ Kg	☐☐, ☐ Kg
13.2	Height (cm)	☐☐ ☐<br>Cm mm	☐☐ ☐<br>Cm<br>mm
For infants aged 0-24 months			
13.3	Name of infant	....	
13.4	Date of birth	____/ _____ /______	
13.5	Weight (kg)	☐☐, ☐<br>Kg	☐☐, ☐<br>Kg
13.6	Height (cm)	☐☐, ☐<br>Cm mm	☐☐, ☐<br>Cm mm

Measuring staff will check the measuring results

***Signature of measuring staff* ____**

***Signature of interviewer* ____**

Thank you very much for participating in this survey

Signature of supervisor* ___________________________*day*_______*month, 2014
