# Supplementary material for: Community support model on breastfeeding and complementary feeding practices in remote areas in Vietnam: implementation, cost, and effectiveness
Source: Int J Equity Health. 2021 May 17;20:121. doi: 10.1186/s12939-021-01451-0 (PMC8127246; doi:10.1186/s12939-021-01451-0)
Supplement: Supplementary file 3 — Additional file 3. A PDF file with Sensitivity data analysis. [file 12939_2021_1451_MOESM3_ESM.pdf]

Additional file 3. Sensitivity data analysis

Additional figure 1. Select breastfeeding (A) and complementary feeding (B) practices by exposure to infant and young child feeding support group (IYCF SG) intervention (INT - A&T IYCF support group assessment survey in 2014). Values are percentages, * differed from comparison communes (CON), † differed from mothers in INT communes, but did not joined support group in the previous three months; $p < 0.05$, two-sided χ^2 test adjusting for clustering. We used data from mothers with children aged 0–23 months for early initiation of breastfeeding, no bottle feeding (n = 1,110); aged 0–5 months for exclusive and predominant breastfeeding (n = 446); children aged 6–8 months (n = 137) for feeding of complementary feeding at 6–8 months; and children from aged 6–23 months (n = 664) for other three complementary feeding practices.

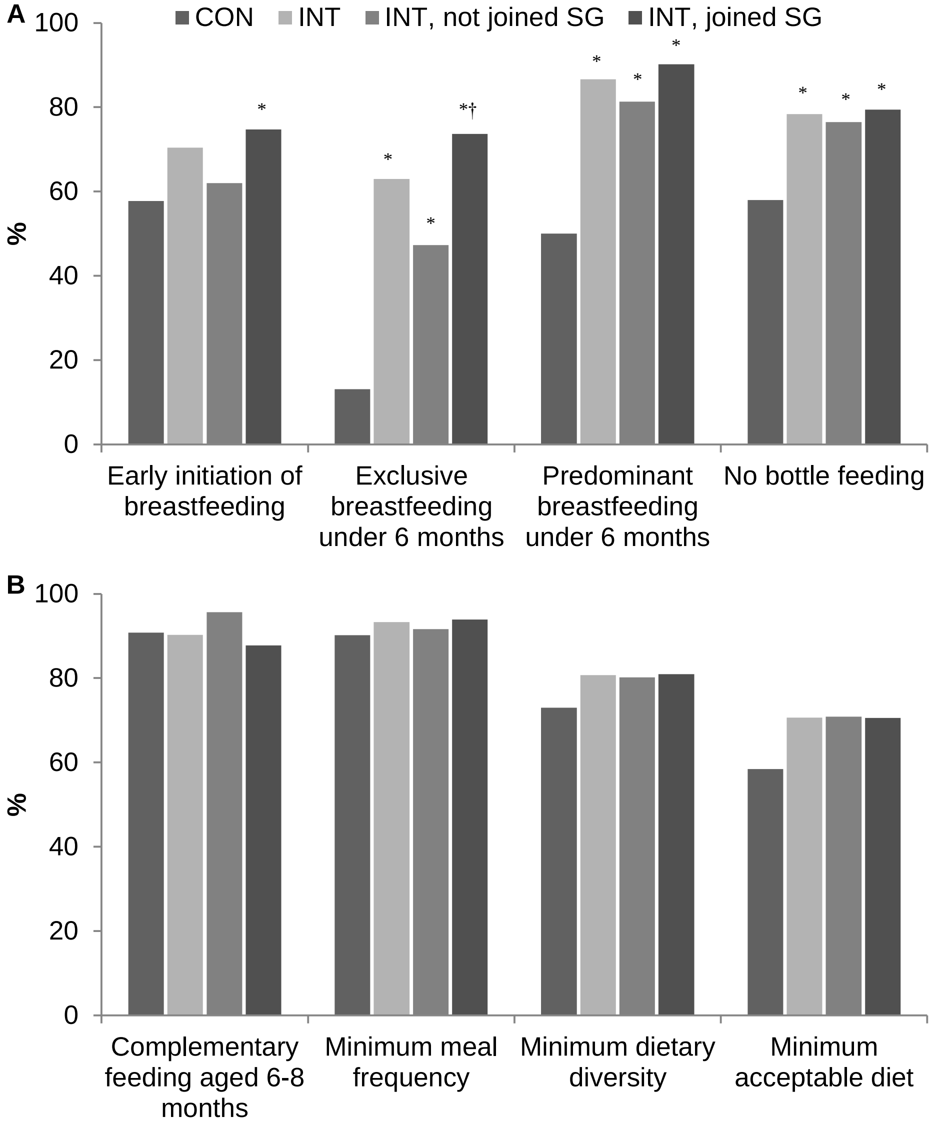

Additional table 1. Association (adjusted OR and 95% CI) between living in a community with an IYCF support group and breastfeeding and complementary feeding practices in mothers with children aged 0–23 months[1]

	Early initiation of breastfeeding (n = 1,110)	Exclusive breastfeeding < 6 months (n = 446)	Predominant breastfeeding < 6 months (n = 446)	Bottle feeding (n = 1,110)	Complementary feeding aged 6–8 months (n = 137)	Minimum meal frequency (n = 664)	Minimum dietary diversity (n = 664)	Minimum acceptable diet (n = 664)
Model 1								
(ref. Comparison communes)								
Living in communes with IYCF support group	1.72*	12.52***	9.64***	2.69***	1.03	1.22	1.33	1.51
	(1.08,2.74)	(6.69,23.44)	(5.07,18.33)	(1.82,3.99)	(0.30,3.55)	(0.65,2.30)	(0.82,2.16)	(0.98,2.33)
Model 2								
(ref. Comparison communes)								
Did not joined IYCF SG	1.18	6.07***	5.74***	2.24**	15.79*	1.03	1.45	1.62*
	(0.75,1.88)	(3.00,12.31)	(2.32,14.19)	(1.31,3.84)	(1.29,193.70)	(0.45,2.37)	(0.81,2.59)	(1.08,2.43)
Joined IYCF SG	2.29***	23.34***	14.88***	2.98***	0.4	1.34	1.28	1.46
	(1.42,3.69)	(11.31,48.16)	(7.41,29.88)	(1.96,4.54)	(0.10,1.60)	(0.64,2.79)	(0.75,2.19)	(0.88,2.44)

[1] Data from A&T IYCF support group assessment survey in 2014. Values are adjusted odds ratios (OR) and 95% CIs, using survey commands to account for clustering. Significantly different from the null value (OR = 1; two-sided t tests): * $p < 0.05$, ** $P < 0.01$, *** $p < 0.001$. Logistic regression models controlled for maternal characteristics (age, ethnicity, education, occupation), household food security, and child characteristics (gender and age). In addition, the also controlled the mode of birth and breastfeeding counseling during pregnancy and at birth.
